# Toward reliable machine learning models for neural circuit inference: A diagnostic study of CNNs on spike trains

**DOI:** 10.1371/journal.pcbi.1014615

**Published:** 2026-08-10

**Authors:** Xiaoqian Sun, Hui Lu, Chen Zeng, Rahul Simha

**Affiliations:** 1 Department of Computer Science, School of Engineering and Applied Science, The George Washington University, Washington, District of Columbia, United States of America; 2 The GW Institute for Neuroscience, The George Washington University, Washington, District of Columbia, United States of America; 3 Department of Pharmacology and Physiology, School of Medicine and Health Sciences, The George Washington University, Washington, District of Columbia, United States of America; 4 Department of Physics, Columbian College of Arts and Sciences, The George Washington University, Washington, District of Columbia, United States of America; Vanderbilt University, UNITED STATES OF AMERICA

## Abstract

Understanding neuronal topology—how neurons are connected—is essential for uncovering neural computation principles and functional organization. However, accurately reconstructing such connectivity remains challenging due to the indirect nature of neural recordings and the complexity of network dynamics. As a first step towards this problem, a growing body of work has explored inferring monosynaptic connectivity directly from spike data. Among these, convolutional neural networks have shown promise when applied to spike-train cross-correlograms. Nevertheless, their ability to generalize across realistic experimental variability and the internal features that drive their predictions remain poorly understood. In this paper, we present a systematic benchmarking and diagnostic study of neural-network-based synaptic inference using simulations across a broad range of biophysical regimes. We show that connectivity classification and synaptic weight estimation, though often combined, rely on distinct internal representations and exhibit markedly different generalization behavior: robust connectivity models emphasize global structure in spike-train correlations, whereas weight estimation models are more sensitive to local signal amplitude and generalize less predictably. Importantly, we find that training on pooled, biologically grounded simulation data substantially improves robustness across parameter perturbations, outperforming models trained under narrow conditions. We further validate these findings in both simulated network data and an *in vitro* dataset from high‑density microelectrode array recordings with patch‑clamp‑verified ground‑truth connections. Models trained on diverse simulated circuits generalize effectively to novel network architectures and the experimental dataset. Together, these results demonstrate that incorporating biologically realistic diversity during training is critical for developing reliable machine-learning tools for large-scale synaptic inference from neural recordings.

## Author summary

Machine learning approaches are increasingly used to infer connectivity from neural activity recordings. In this study, we take a step back from proposing new algorithms and instead focus on understanding the reliability of existing machine-learning models for synaptic inference. By systematically testing models across a wide range of biologically realistic circuit conditions, we identify factors that limit their ability to generalize. We show that models trained on narrow or idealized data often develop biases, whereas training on diverse, biologically grounded data substantially improves robustness. We further demonstrate that this strategy enables models trained on simulations to generalize to experimental recordings with known connections, providing practical guidance for building more reliable tools for mapping neural circuits.

## Introduction

The architecture of neuronal circuits underlies their ability to process information, respond to external stimuli, and generate behaviors. Mapping synaptic connectivity from neuronal activity is an important first step towards bridging the gap between microscopic wiring and macroscopic function. Once connectivity is reliably known, the dynamics of such connected neurons can then provide critical insight into how behavior and cognition arise [1,2]. While anatomical reconstructions reveal the structural layout of connections, they rarely inform how strongly or dynamically neurons communicate [3–5]. Conversely, functional correlations between spike trains or calcium traces often reflect indirect or shared-input effects [6,7]. What we ultimately seek to infer is monosynaptic connectivity, a direct influence between neurons measured in units of excitatory or inhibitory postsynaptic potentials (EPSPs/IPSPs) [8]. Directly measuring monosynaptic connectivity via intracellular recordings provides the most rigorous ground truth but remains technically limited to a small number of neurons and largely restricted to *in vitro* preparations [9]. In contrast, extracellular recording techniques—such as high-density microelectrode array (HD-MEA), Neuropixels probes, and calcium imaging—can simultaneously capture activity from hundreds to thousands of neurons but do not directly resolve synaptic contacts. Consequently, inferring monosynaptic connectivity from spike trains has become a central analytical goal, enabling researchers to reconstruct circuit-level wiring and synaptic strength from large-scale population recordings [10].

Since the 1970s, spike-to-spike cross-correlations (CCGs) have been used to infer synaptic connectivity by identifying narrow peaks or troughs within a few milliseconds around zero time lag, where zero denotes synchronous spiking between a putative pre- and postsynaptic neuron [11]. In recent years, more than half of all studies of monosynaptic connection inference have been based on co-spiking correlations, and roughly a quarter employ CCGs[10]. Yet, CCGs, far from being static stationary signals, are highly variable and are shaped by biological and experimental factors [12], excitatory/inhibitory balance [13], background synaptic noise, connectivity density [14], bursting activity [14,15], synaptic connection strength [16], circuit activity level [17] and recording duration [18], all of which can distort or mask synaptic signatures. Over decades, refinements such as jitter correction [19,20], statistical decomposition [15,16] and post-processing [21,22] have been proposed.

In recent years, machine learning techniques have expanded the set of inference tools available for this problem. Donner, et al. [23] induced an ensemble artificial neural network framework (eANN), which integrates connectivity scores and matrices derived from six methods, including coincidence index (CI), smoothed cross-correlogram (sCCG), directed spike time tiling coefficient (dSTTC), generalized linear model cross-correlogram (GLMCC), transfer entropy (TE) and generalized linear model point process (GLMPP), into a multi-layer perceptron that jointly infers the presence and strength of monosynaptic connections. Although eANN improves inference by integrating multiple model-based metrics, it inherits the parameter sensitivity of its model-based inputs, which must be tuned for each dataset. Endo, et al. [18] proposed a convolutional neural network (CNN) model (CoNNECT) which bypasses per-dataset parameter tuning and directly learns from raw CCGs. Though CoNNECT achieved competitive performance on both simulated and experimental data and illustrated the temporal patterns captured by its learned convolutional filters, the study primarily evaluated global metrics such as Matthews correlation coefficient (MCC) and false-positive/negative rates, offering limited insight into the internal feature representations that underlie the CNN’s decisions. Moreover, robustness analyses were restricted to a narrow set of conditions, including neuronal models, recording durations, channel counts and simple time-rescaling, leaving open the question of how well the model generalizes across biological variability, parameter perturbations, or domain shifts. Despite decades of effort, the field still lacks a mechanistic understanding of why modern machine-learning models succeed or fail in synaptic inference. Prior work has emphasized performance benchmarks rather than examining how CNNs internally encode CCG structure, how biological variability disrupts these encodings, or how generalization might be systematically improved. Although it may seem intuitive that inference models require training diversity, this principle has not previously been demonstrated, quantified, or linked to the internal failure modes of neural networks. We introduce the first comprehensive perturbation and diagnostic framework for CNN-based synaptic inference, revealing that CNN failures arise from representational biases—such as amplitude over-reliance and unimodal feature-map collapse—rather than from experimental noise alone. We further show that training on biologically diverse simulations yields robust, transferable models that generalize to unseen network architectures and experimental HD-MEA recordings. These findings transform synaptic inference from a black-box machine-learning task into a mechanistically interpretable and principled pathway toward reliable large-scale circuit reconstruction.

In this study, we performed a systematic benchmarking and diagnostic analysis of CNN-based monosynaptic inference. Using a comprehensive suite of biologically grounded simulations, we quantify CNN performance and generalization across parameter-perturbations in excitatory-inhibitory ratio, bursting dynamics, varying synaptic weights and background noise. We show that connectivity classification and synaptic-weight regression depend on distinct internal CNN representations, with the former emphasizing global signal organization and the latter relying more on combinational cues. Motivated by the observations that weak models over-rely on absolute signal amplitudes, we further evaluate CNNs trained on amplitude-normalized CCGs. Although this scaling improved robustness under controlled perturbations, models trained on raw CCGs generalize better across heterogeneous domains. Finally, by training CNNs on pooled CCGs from multiple simulated networks and testing them on a topologically distinct unseen network as well as an *in vitro* HD-MEA dataset with patch-clamp-verified connections, we demonstrate that models trained on diverse simulated circuits generalize effectively to real neuronal systems.

Together, these results highlight the intricate interplay between biophysical parameter variability, CCG signal structure, and neural-network inference, providing a foundation for reliable large-scale synaptic reconstruction from experimental data.

## Results

### Synthetic data pipeline and CNN optimized performance

**Fig 1:**
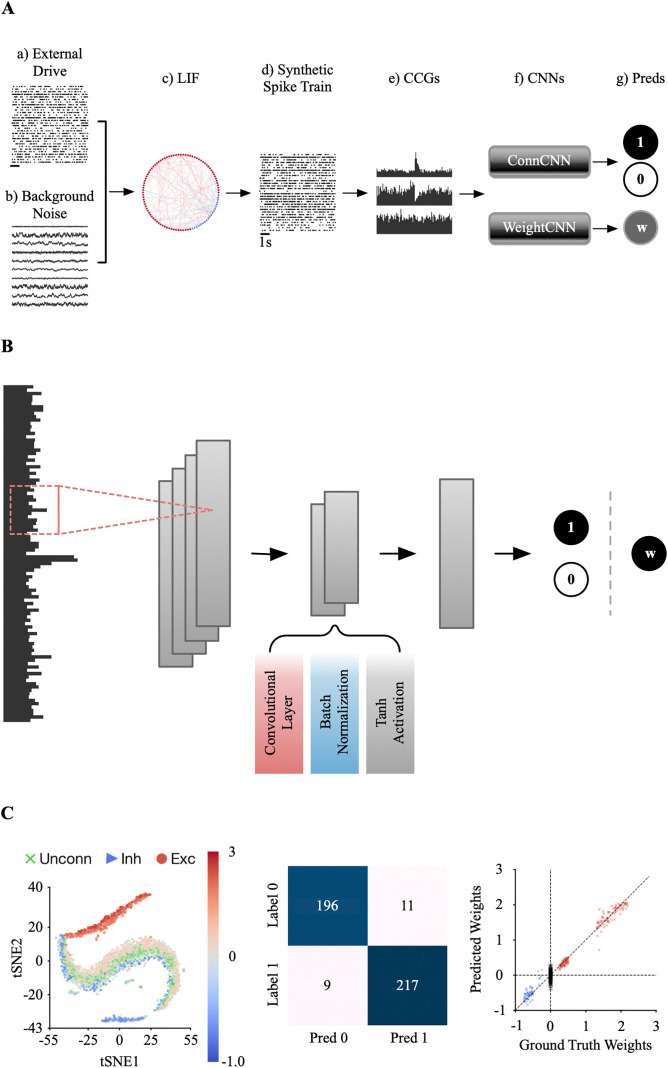
Synthetic data pipeline and optimized performance of CNN models. **(A)** End-to-end workflow for synthetic data generation and inference framework. A recurrent leaky integrate-and-fire (LIF) network was simulated under stochastic drive from external sources with stochastic bursting activity and Ornstein-Uhlenbeck background noise. Pairwise cross-correlogram (CCGs) were computed from simulated spike trains and labeled with ground-truth connectivity and synaptic weight, forming the dataset for model training. **(B)** CNN architecture. Two complementary CNNs were trained on CCG inputs: ConnCNN for binary classification of connection existence and WeightCNN for continuous regression of synaptic strength. The optimized architecture consists of two convolutional blocks (each containing a convolutional layer, batch normalization and Tanh activation), followed by an average pooling, a dropout, and a fully connected output layer. Filter activations were studied in subsequent analysis. **(C)** Optimized model performance. Left: t-SNE embedding of CCGs colored by synaptic weight (unconnected - green crosses, excitatory - red dot, inhibitory - blue triangle). Middle: confusion matrix showing accurate connection classification (95.38% accuracy on test dataset). Right: Weight regression plot demonstrating strong correspondence between predicted and ground-truth synaptic weight (MSE = 0.008).

To establish a framework for data-driven synaptic inference, we first built a complete pipeline linking neuronal network simulation, CCG generation, and CNN inference (Fig 1A). Following previous work [15,24], we simulated a recurrent network of 100 leaky integrate-and-fire (LIF) neurons composed of 80 excitatory and 20 inhibitory cells (**see Methods**). Each neuron received stochastically driven input from 10 other excitatory and 10 other inhibitory neurons exhibiting random bursting activity, along with background pink noise (weighted towards lower frequencies) modeled by an Ornstein-Uhlenbeck process. Pairwise raw cross-correlograms (CCGs) were computed from the synthetic spike trains and paired with ground-truth labels indicating connection presence and synaptic weight for the purpose of training CNNs. For each presynaptic neuron, CCGs were calculated with both connected postsynaptic partners and an equal number of randomly selected unconnected neurons (firing rate > 0.5 Hz for both groups). The resulting dataset was randomly shuffled and partitioned into training, validation, and test subsets in a 70:15:15 ratio, while maintaining the same proportion of excitatory and inhibitory connections across subsets through stratified sampling.

Raw CCGs served as inputs to two convolutional neural networks with complementary objectives, ConnCNN, which classifies connection existence (binary output), and WeightCNN, which regresses synaptic strength. The synthetic dataset served as a controlled platform for tuning the CNN architecture and hyperparameters, enabling systematic model selection and performance evaluation (**see Methods**). After extensive optimization, the final CNN architecture comprised two convolutional blocks, each containing a convolutional layer, batch normalization, and Tanh activation, followed by average pooling, dropout, and a fully connected output layer (Fig 1B).

The performance of the optimized CNN models is summarized in Fig 1C. t-distributed stochastic neighbor embedding (t-SNE) visualization shows CCGs clustering (left), where each point represents a CCG from a neuron pair and is colored by its synaptic weight. CCGs correspond to unconnected pairs (gray crossings) intermingle with weakly weighted synapses (light red or blue hues). The ConnCNN confusion matrix (middle) indicates accurate binary discrimination (95.38% test accuracy), and the WeightCNN regression (right) reveals a close correspondence between predicted and ground-truth synaptic weights (mean squared error MSE = 0.008). Together, these results establish a robust CNN architecture and performance reference for the subsequent systematic benchmarking and interpretability analysis.

### Illustration of the controlled benchmarking of CNN robustness across biophysical perturbations

**Fig 2:**
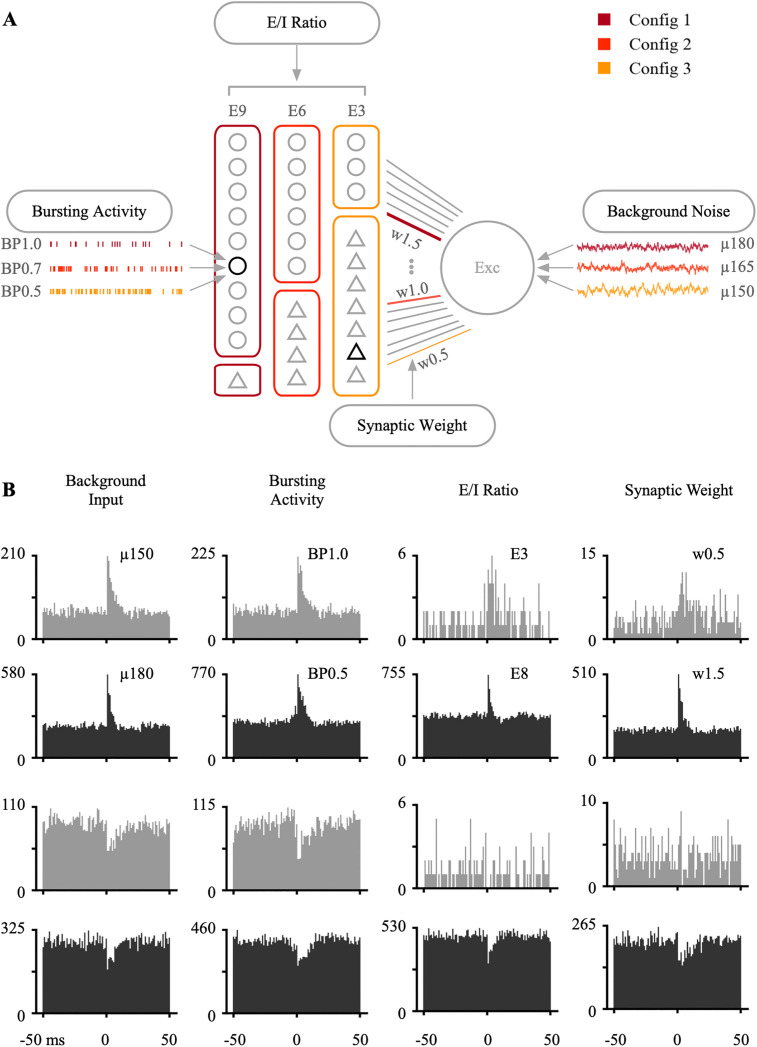
Systematic benchmarking framework for CNN-based synaptic inference. **(A)** Schematic overview of the controlled benchmarking design. Each simulated circuit consists of 40 presynaptic neurons projecting to a single postsynaptic neuron. CNN robustness was evaluated under systematic perturbations of four biophysically meaningful parameters: the mean level of background noise to the postsynaptic neuron (μ), presynaptic bursting probability (BP), the excitatory–inhibitory ratio (E/I) of presynaptic neurons, and synaptic conductance scaling (w). For each parameter category, three representative example configurations (Config 1-3, color-coded) are shown to illustrate how distinct parameter regimes give rise to quantitatively different spiking and CCG patterns. For bursting activity, lower BP values produce longer burst sequences, whereas BP=1.0 corresponds to non-bursty Poisson-like spiking. For background input, increasing μ elevates the overall fluctuation level of the postsynaptic drive. E/I perturbations modify the proportion of excitatory and inhibitory presynaptic neurons, while synaptic weight perturbations scale the strength of presynaptic inputs to the postsynaptic neuron. See S1 Table for full baseline and perturbation parameter sets. **(B)** Representative excitatory and inhibitory CCGs generated under controlled parameter regimes for the four perturbation axes used in the benchmarking framework. Each column corresponds to one perturbation axis. Within each column, the upper two rows show excitatory CCGs and the lower two rows show inhibitory CCGs. Gray indicates low-end parameter settings in each axis (e.g., μ150, BP1.0, E3, w0.5), and black represents high-end settings (e.g., μ180, BP0.5, E8, w1.5). All CCGs were computed within a ± 50 ms window.

To systematically evaluate the robustness of CNN-based connectivity inference, we designed a suite of controlled benchmarking experiments (Fig 2A). Here, we use a simplified feedforward circuit consisting of 40 presynaptic neurons projecting to a single postsynaptic neuron. These tests probe when and how model performance deteriorates as circuit biophysics deviate from the conditions seen during training. Specifically, we examined four key parameters that shape neural dynamics and CCG structure: i) the mean level of background pink noise to the postsynaptic neuron (μ), ii) the bursting probability of presynaptic inputs (BP; higher BP corresponds to shorter bursts, with BP=1.0 indicating no bursting; **see Methods**), iii) the ratio of excitatory to inhibitory presynaptic neurons (E/I), and iv) the overall synaptic weight strength (w).

**Fig 3:**
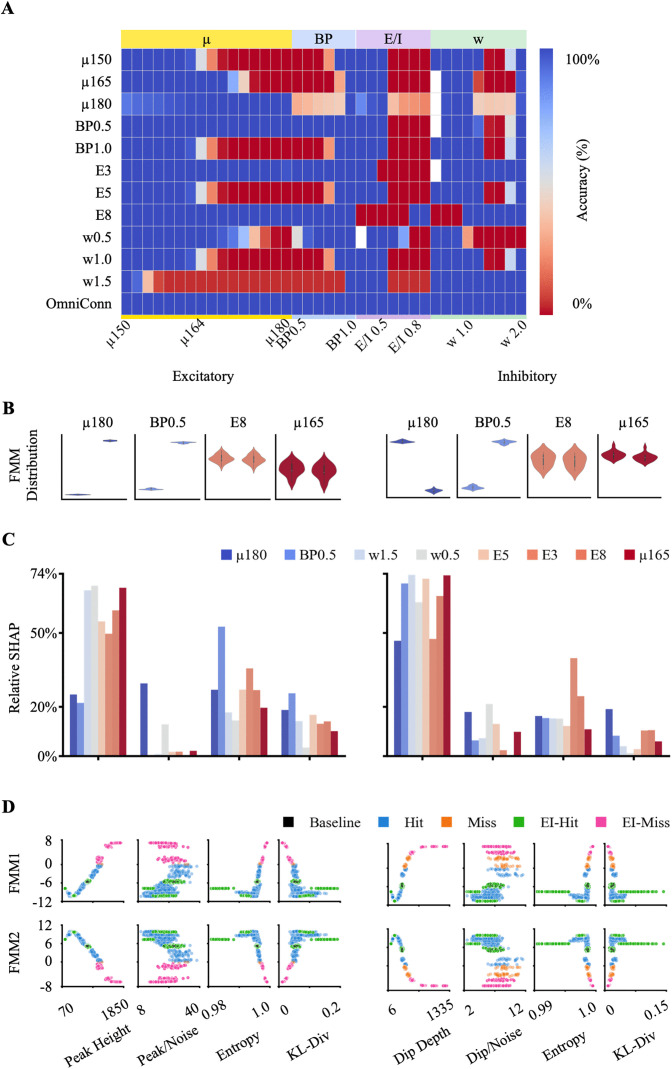
Connectivity-inference benchmarking and CNN representation analysis. **(A)** Mean accuracy over fifty CNN training and testing iterations. Each row represents a baseline model performance on perturbations, and the last row is that of CNN model trained on all baseline datasets. Four groups of perturbations, **i)** μ group, μ changing from 150 to 180 with increment of 2; **ii)** BP group, BP incrementing from 0.5 to 1.0 with step = 0.1; **iii)**, E/I group with varying E/I ratio from 0.3 to 0.9; **iv)** w group, scaling weights increasing from 0.4 to 2.0 with stride = 0.2. **(B-D)** CNN model internal representation analysis. **(B)** Feature map mean (FMM) distribution for excitatory (left) and inhibitory (right) connections for four representative baseline models (μ180, BP0.5, E8 and μ165). FMM values represent globally averaged feature-map activations in the last convolutional layer; larger absolute FMM values indicate stronger activation of learned CNN feature detectors. For visual clarity, only the relative distribution structure of the FMMs is emphasized in the main figure; full FMM axis scales and quantitative distributions are provided in S2 Fig. **(C)** SHAP feature attribution of three indicators (peak height/dip depth, normalized entropy and KL divergence of CCG 10ms around time lag 0 ms). Higher SHAP values indicate stronger contribution of the corresponding indicator to the CNN prediction. **(D)** Scatter plots of FMMs against four indicators for the baseline model BP0.5. Each point represents one sample: black dots representing samples from training baseline dataset, green and magenta dots marking samples from E/I perturbations, while blue and orange dots marking samples from all other perturbations. Clear separation between correct and incorrect predictions indicates that the learned feature representations are strongly associated with underlying CCG structure.

**Fig 4:**
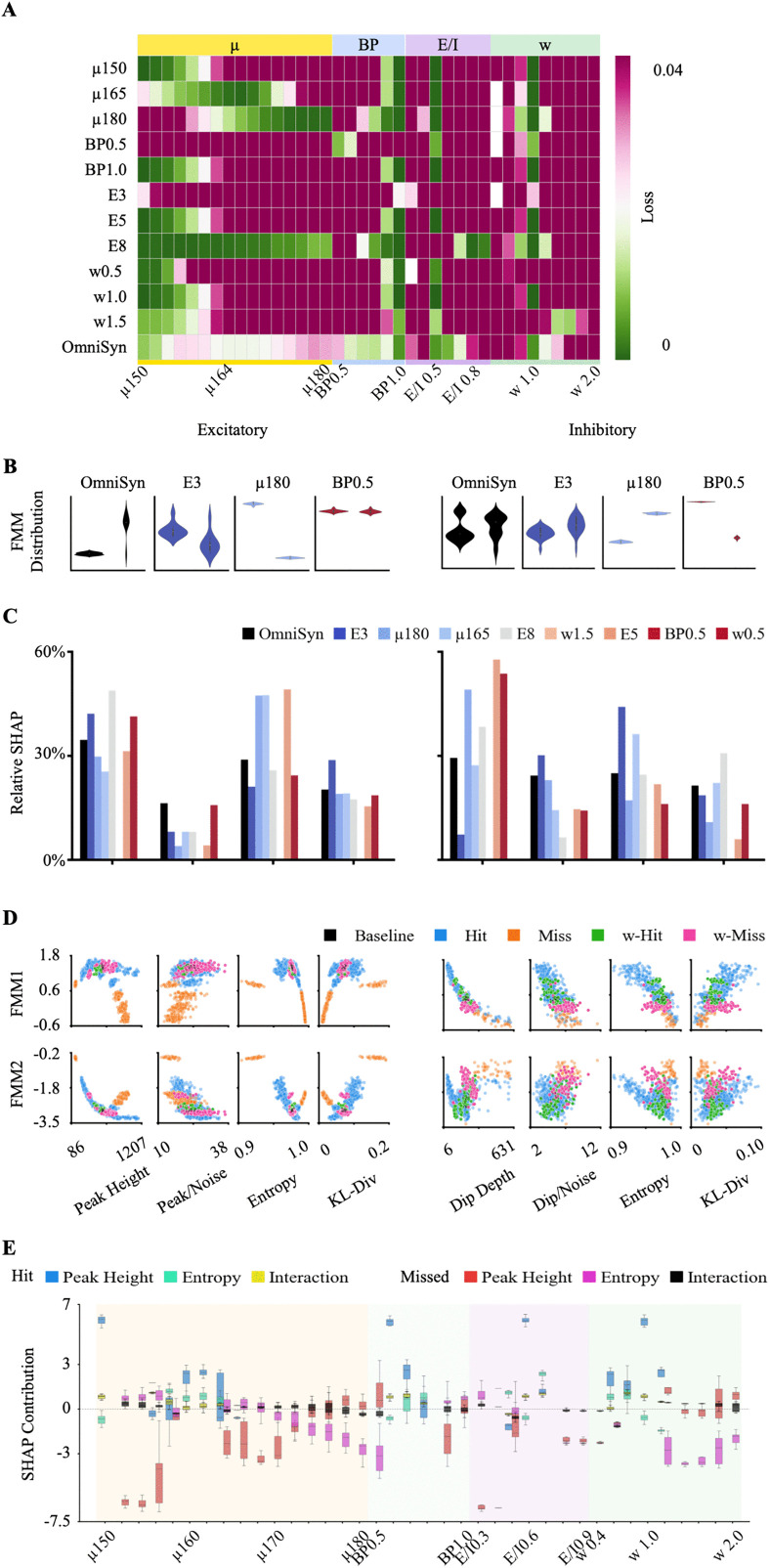
Weight-inference benchmarking and CNN representation analysis. **(A)** Mean loss over fifty CNN training and testing iterations. Similar to Fig 3A except for the color scheme and scale. For weight inference, we set the upper limit of loss to be 0.04, 20% deviation of the ground truth. **(B-D)** CNN model internal representation analysis. Similar to Fig 3B-3D. RF was able to be trained on OmniSyn model since it had a moderate number of “missed” cases where MSE > 0.04. **(B)** Feature map mean (FMM) distribution for excitatory (left) and inhibitory (right), **(C)** SHAP feature attribution, and **(D)** scatter plots of FMMs against four indicators for the baseline model μ180. **(E)** SHAP interaction analysis of the μ180 excitatory XGBoost model. Shown are individual and joint SHAP contributions for peak height and entropy, within interaction strengths summarized as box plots across perturbations. The background color denotes the perturbation group, light yellow - μ perturbation, light blue - BP perturbation, light purple - E/I perturbation, and light green - w perturbation.

Each simulation consisted of a minimal circuit of 40 presynaptic neurons projecting to one postsynaptic neuron, enabling fine control over single-parameter perturbations. For each circuit, positive samples corresponded to CCGs between each presynaptic neuron and the postsynaptic neurons, while negative samples were generated by pairing each presynaptic neuron with a randomly selected presynaptic neuron. To reduce sampling variability, each circuit configuration was simulated five times with different random seeds and CCG samples from all simulations were pooled to form the corresponding training or testing dataset. For each parameter, we generated a baseline dataset with a fixed parameter combination (e.g., μ=150, BP=1.0, E/I=0.5,w=1.0), trained a CNN on that baseline dataset, and then tested it on systematically perturbed datasets in which only one parameter was varied while others were held constant. For example, in the μ-section, μ was varied from 150 to 180 in steps of 2, while BP, E/I, and w remained fixed; analogous perturbation sweeps were performed for BP (0.5−1.0), E/I (0.3−0.9), and w (0.4−2.0). We evaluated 10 baseline configurations in total (e.g., μ150, μ165, μ180, BP0.5, BP1.0, E3, E5, E8, w0.5, w1.0, w1.5), each trained and tested over 50 independent seeds to ensure statistical reliability. Representative excitatory and inhibitory CCGs under baseline regimes are shown in Fig 2B. Consistent with these examples, the perturbations produced distinct distributions of CCG statistics and different training regimes therefore exposed the CNN models to substantially different signal structures (S1 Fig). The resulting accuracy and loss matrices quantify how performance degrades under controlled perturbations, allowing us to dissociate the impact of biophysical variability from that of model inductive biases. Note that the baseline model μ150, BP1.0, E5, and w1.0 were identical in training data and parameter setting; these conditions were therefore collapsed into the E5 baseline for SHapley Additive exPlanations (SHAP, **see Methods**) analysis. Full comparisons are shown in Figs 3A and 4A, and S1 Table summarizes the parameter sets and perturbation ranges from all benchmark experiments. For each baseline configuration, the model achieving the lowest mean perturbation loss across all test conditions was selected as the representative instance for subsequent diagnostic analyses, as it exhibited the most robust generalization behavior. Population-level statistics (e.g., mean accuracy and loss) were computed across all runs to provide a comprehensive assessment of performance variability.

### Diagnostic analysis of CNN connectivity inference across perturbations

The classification accuracy of ConnCNN across all perturbation tests is summarized in Fig 3A. Three major patterns emerge: i) baseline models generalized well near the training regime, e.g., mu150 achieved high accuracy (blue blocks) within μ∈[150, 162], BP∈[0.9, 1.0], E/I ∈[0.3, 0.5] and w∈[0.4, 1.2]; ii) none of the models generalized broadly across the E/I section; iii) generalization behavior depends on specific parameter and training regime rather than following a simple monotonic trend. For example, μ180, trained under higher background input, showed broader generalization across multiple perturbation axes; w1.5, trained under stronger synaptic weights, showed improvement mainly along weight-related perturbations; and, BP0.5, trained with more bursting activity, improved generalization across μ and BP perturbations but not across E/I or weight perturbations. These results suggest that exposure to broader training regimes can promote the learning of features that generalize more effectively to certain unseen conditions, although this effect is parameter-dependent and not uniformly observed across all perturbations.

To quantitatively describe model robustness and signal structure, we introduced two measures: a robustness ranking score and a set of CCG indicators. The ranking score (**see Methods**) was defined as the harmonic mean of accuracies on self- and cross-perturbation tests, with high values indicating better overall performance. The top-ranked baselines were μ180, BP0.5, w1.5 (see S2 Table for a complete summary of model ranking metrics). Notably, these top-performing models correspond to regimes with more heterogeneous and higher-variance activity patterns, supporting the observation that such training conditions can enhance generalization, although the effect remains uneven across all perturbations. Each model in Fig 3B-3C is color-coded by this score (blue-robust, red-fragile). CCG indicators were designed to capture both local and global signal attributes, including peak or dip amplitude, width, lag, entropy, and KL divergence, providing a quantitative description of CCG morphology (**see Methods**).

To identify the origins of performance variation, we conducted a three-step diagnostic analysis: i) we examined feature-map mean (FMM) distributions in the last convolutional layer to characterize internal representations; ii) we trained a Random-Forest (RF) model using CCG indicators to predict whether each CCG input was correctly classified, and used SHAP analysis to quantify indicator contributions to CNN decisions; iii) we related internal activations to input statistics by plotting FMM values against key indicators across samples. Here, FMM (**see Methods**) refers to the globally averaged activation of each feature map (channel) in the final convolutional layer after pooling, providing a low-dimensional summary of the network’s learned representation for each input. Higher absolute FMM values therefore indicate stronger activation of the learned convolutional filters in response to a given CCG pattern. In our model architecture, this results in two scalar values per sample (FMM1 and FMM2), corresponding to the two output channels of the last convolutional block. Together, these analyses form a closed-loop link between model performance, internal representation, and CCG structure.

As shown in Fig 3B, models with higher robustness scores exhibited bipolar FMM distribution (excitatory-left, inhibitory-right), while weakly generalized models showed unimodal FMMs, indicating reduced feature differentiation (see S2 Fig for all baseline model FMM distribution). This bipolar organization suggests that successful models learned more separable internal representations for different CCG structures. SHAP analysis (Figs 3C; S3) revealed that, for both excitatory (left) and inhibitory (right) CCGs, models with stronger generalization attenuated reliance on absolute deflection magnitude and distributed their reliance across complementary statistical cues (peak/dip-to-noise, normalized entropy, and local KL divergence). Note that higher SHAP values indicate that the corresponding indicator contributes more strongly to the CNN prediction. This multi-feature integration, most pronounced in the μ180 (OmniConn in S3 Fig), indicates that successful inference arises from integrating amplitude, variability and temporal information content, rather than from simple deflection magnitude alone. Finally, we examined the BP0.5 baseline model’s behavior across perturbations to understand why the model failed to generalize to other E/I conditions (Fig 3D; see also S4 Fig for all baseline models). This panel shows scatter plots of FMM against four indicators (peak height/dip depth, peak/dip-to-noise, normalized entropy, and KL divergence of the focused window) and samples from E/I perturbation series are highlighted in green (correct) and magenta (missed). Across all indicators, a clear separation emerged between correctly and incorrectly predicted samples and FMM showed a strong positive correlation with peak height, FMM increasing linearly with peak height in excitatory pairs and following an exponential-like saturation in inhibitory pairs. Once peak height exceeded a certain threshold and FMM values crossed zero toward the positive side, predictions consistently failed, delineating a clear decision watershed between correct and incorrect predictions. Lower entropy and higher KL divergence indicate a more pronounced and localized deflection in the CCG, reflecting stronger temporal coupling between neuron pairs. Although peak height kept increasing, peak-to-noise level showed a retrograde trend, accompanied by high entropy and lower KL divergence. This pattern suggests that elevated background correlations, driven by globally increased network firing rates, broaden the CCG profile, reducing its relative structure despite the larger absolute peak amplitude. Together, these analyses indicate that CNN generalization failures stem from over-dependence on absolute signal strength rather than relational structure within the CCG. Notably, the two feature-map outputs (FMM1 and FMM2) exhibit approximately mirrored (anti-correlated) structures across all indicators. This reflects that the two convolutional channels have learned complementary feature detectors, capturing opposing aspects of the CCG signal. As a result, the network effectively represents each input along a low-dimensional bipolar axis defined by these opposing features. This representation enables clear separation between correctly and incorrectly classified samples, but also makes the model sensitive to shifts in signal amplitude, contributing to the observed generalization failures under E/I perturbations.

### Pooled training further improves ConnCNN model generalization

Inspired by the observation that CNNs trained with richer or more heterogeneous datasets exhibit improved generalization, we trained an additional model, OmniConn, on the union of all baseline datasets and evaluated its performance across all perturbations (**last row in** Fig 3A). Unlike individual baseline models, OmniConn, trained on pooled data, maintained near-perfect accuracy (uniform blue blocks) across all perturbation domains, demonstrating that CNNs can generalize when exposed to sufficiently diverse training conditions. SHAP analysis on RF trained on pooled inhibitory indicators to predict OmniConn outputs showed that the pooled model relied less on dip depth compared with even the best single-baseline model μ180 (S3 Fig). This result confirms that the poor generalization of single-baseline models stems from representation bias rather than architectural limitation.

### Diagnostic analysis of CNN-based synaptic weight inference across perturbations and the effect of pooled training

We next applied the same benchmarking framework to the CNN trained to infer synaptic weights (WeightCNN). The regression performance across perturbations is summarized in Fig 4A, where the mean-squared error (MSE) upper limit was set to 0.04, corresponding to a maximum deviation of 20% from the ground-truth weight. Model robustness scores were calculated as the harmonic mean of self- and cross-perturbation hit rates (defined as MSE < 0.04), with higher scores indicating better generalization.

Compared with ConnCNN, WeightCNN exhibited narrower generalization ranges. For instance, the baseline model μ150 achieved MSE < 0.02 (blue blocks) only within μ∈[150, 158], BP∈[0.9, 1.0],E/I=0.5 and w=1.0. Only one model, E8, generalized across μ-perturbations, while all baselines failed to generalize broadly across any parameter perturbations. Unlike ConnCNN, no consistent relationship was observed between parameter magnitude and generalization performance. Models trained on high-end parameter settings did not necessarily generalize better, suggesting that weight inference is less tolerant to parameter shifts and lacks the robustness observed in binary connectivity classification.

Diagnostic analyses revealed several key distinctions from ConnCNN. First, bi-polar FMM distributions in Fig 4B were not consistently associated with robust models, indicating weaker feature selectivity for weight regression. Second, SHAP analysis (Fig 4C) showed that the contribution gap between absolute amplitude cues and global statistical cues was smaller, implying that WeightCNN integrates multiple signal types in a less hierarchical manner. Finally, visualizing FMMs against CCG indicators (Fig 4D; see also S5 Fig for all baseline models) across w perturbations showed substantial overlap between correctly predicted samples (blue) and misclassified samples (orange), in contrast to the clear separability observed in connectivity inference (Fig 3D). In addition, unlike the connectivity models, the two feature-map outputs (FMM1 and FMM2) do not exhibit a clear anti-correlated or mirrored structure. This suggests that the network does not learn a simple bipolar representation but instead relies on a more entangled combination of features. These results suggest that weight estimation might rely on combinatorial feature integration rather than isolated cues.

To further probe feature interactions, we trained an Extreme Gradient Boosting (XGBoost) model using CCG indicators to predict whether a CCG’s synaptic weight was accurately inferred and analyzed SHAP interactions between indicator pairs. Fig 4E shows results from the μ180 baseline model, visualizing individual SHAP contributions of the two most influential indicators, peak height and entropy, as well as their pairwise interactions, summarized as boxplot across perturbations. Successful predictions tended to occur in regions where peak height and entropy contribute in a coordinated way, whereas missed predictions were enriched where interactions were weak or dominated by a single cue. In some perturbations, positive SHAP contributions of entropy coincided with negative peak height effects, while the opposite held true in some others, reflecting compensatory dynamics between signal irregularity and amplitude cues in the models’ decision process.

Following the same approach as for OmniConn, we trained OmniSyn on the union of baseline datasets and evaluated it against the pooled perturbation set. Although OmniSyn did not achieve the same level of performance as OmniConn, it demonstrated markedly improved stability across conditions. As shown in the last row of Fig 4A, extreme MSE values (red blocks) largely disappeared, and performance remained moderate (light blue or red) throughout. While it did not minimize loss for every individual perturbation, OmniSyn avoided catastrophic failures and maintained consistent performance across diverse scenarios. This underscores the importance of broad training distributions in enhancing model robustness and reliability under biophysically variable conditions.

Across runs, we also quantified seed-to-seed variability using the coefficient of variation (CV) across 50 training instances. ConnCNNs were highly stable (CV<0.15 on average), with only μ180 and w0.5 baselines showing noticeable dispersion. In contrast, WeightCNNs exhibited much higher variability (CV ∈[0.25, 4.5]), indicating greater sensitivity to random initialization and training noise. Importantly, pooling across baseline domains markedly suppressed this variance, with the pooled WeightCNN displaying the most consistent performance across perturbations (S6 Fig).

Across both tasks, SHAP consistently ranked peak height or dip depth as the most influential indicator, even when robust ConnCNNs de-emphasized amplitude relative to entropy or KL divergence. This amplitude bias motivated a scaled-CCG analysis to reduce reliance on absolute magnitude and test whether generalization improves without changing architecture.

### Amplitude-normalized benchmarking reveals trade-offs between robustness and signal fidelity

To test whether CNNs’ sensitivity to absolute CCG amplitude could be mitigated without explicit multidomain retraining, we applied tail-reference normalization to all CCGs prior to training (**see Methods**). This normalization rescales each CCG relative to fluctuations in the outer time-lag regions, reducing dependence on raw peak/dip magnitude while preserving temporal structure.

**Fig 5:**
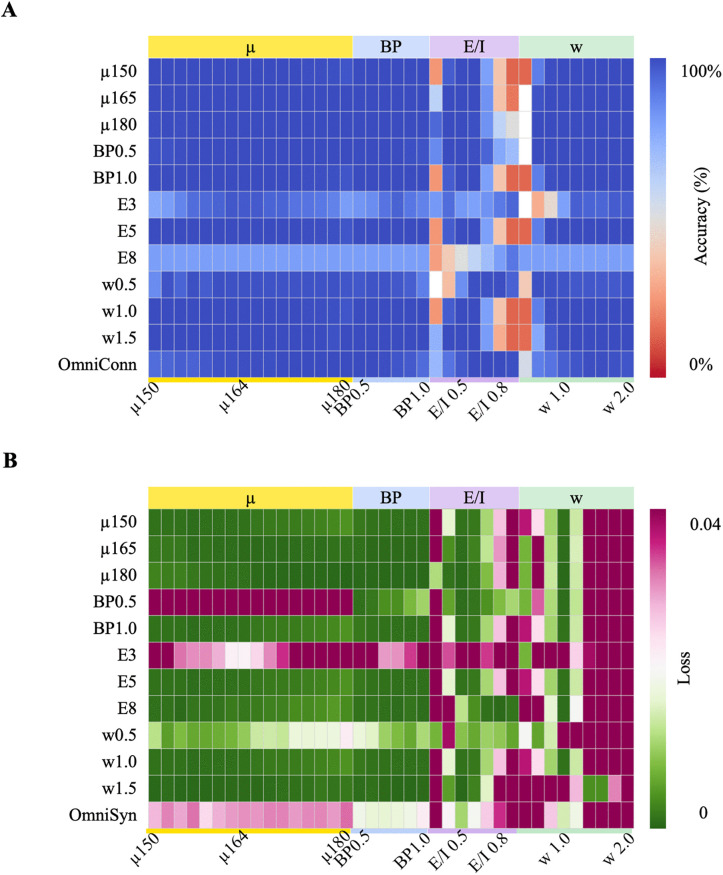
Average performance of CNNs trained on amplitude-normalized CCGs across perturbations. **(A)** Mean accuracy of baseline models for connectivity inference trained on amplitude-scaled CCG datasets, analogous to Fig 3A. **(B)** Mean loss of WeightCNN models trained on scaled CCGs, analogous to Fig 4A. Color maps represent model performance across baseline-to-perturbation testing conditions, with purple indicating failure zones and green indicating stable generalization.

In the connectivity task (Fig 5A), ConnCNN models trained on scaled CCGs exhibited markedly improved generalization across all perturbations. While performance in the E/I-perturbation section remained reduced, large-scale failures (red blocks) were largely eliminated, replaced by mild degradation (orange). In the weight-inference task (Fig 5B), scaled WeightCNN models similarly achieved lower and more stable mean losses across perturbations, particularly within μ- and BP- sections. However, performance still declined for strong-conductance perturbations (w>1.4), except for model trained at w=1.5.

Scaling improved stability within single domains but reduced cross-domain generalization. As shown in the last row of Fig 5A and 5B, OmniConn and OmniSyn trained on scaled CCGs underperformed their raw counterparts. When trained on pooled raw baseline data, OmniConn achieved near-perfect generalization across all perturbations, whereas the version trained on pooled scaled data exhibited mild degradation. Likewise, the pooled scaled OmniSyn showed consistent but higher loss, with light-blue regions in the raw model shifting toward light red.

Together, these results demonstrate that amplitude normalization enhances robustness in controlled perturbations but attenuates informative signal structure carried by absolute CCG magnitude. While scaling mitigates overfitting to amplitude, it might also remove biophysically meaningful cues related to synaptic strength and relative amplitude contrast, suggesting that amplitude carries both nuisance variability and task-relevant information.

### Global firing-rate modulation reveals sensitivity to activity-distribution shifts

To further evaluate whether the observed CNN robustness extends beyond stationary perturbation regimes, we introduced temporally varying global firing-rate modulation into the 40 → 1 benchmarking framework. Unlike previous perturbations in which presynaptic firing rates remained constant throughout the simulation, here presynaptic firing rates changed across successive time periods within the same simulation while the circuit connectivity and synaptic parameters remained unchanged. Specifically, we used the E5 configuration (BP=1.0, E/I=0.5, μ=150, w=1.0) and generated Poisson spike trains with piecewise-constant firing rates, where each firing-rate level persisted for the same simulation duration. We generated four modulation regimes with progressively increasing temporal variability while maintaining the same mean firing rate across the full simulation: i) Base (constant 20 Hz), ii) Mild modulation (15–20–25 Hz), iii) Moderate modulation (10–20–30 Hz), and iv) Strong modulation (5–15–25–35 Hz). An additional Mean-shifted modulation regime (5–15–30–45 Hz) was introduced to simultaneously increase both modulation strength and overall firing-rate statistics. Representative CCGs from the five modulation regimes and distributions of CCG indicators are shown in S7 Fig. The CCG indicators distributions showed systematic shifts across modulations: peak height (dip depth), normalized entropy and KL divergence exhibited progressively displaced distributions from Base to Strong modulation, with partial overlap between neighboring regimes, whereas the Mean-shifted condition formed a substantially separated distribution with minimal overlap, while peak/dip to noise ratio distributions remained highly overlapping across all modulation regimes.

**Fig 6:**
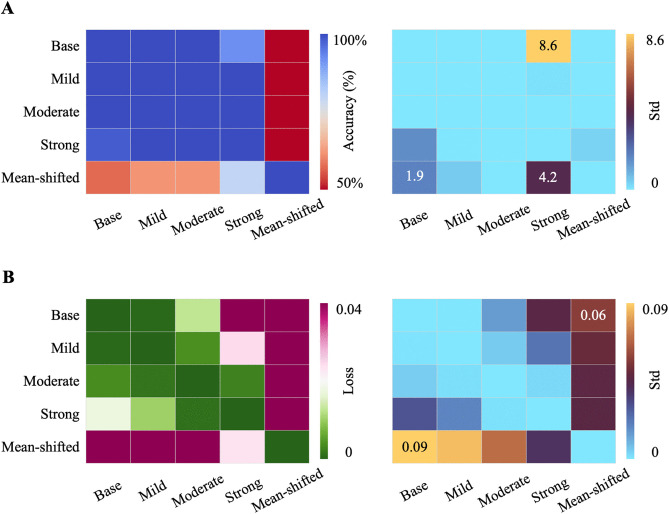
Robustness of connectivity and weight inference under temporally varying global firing-rate modulation. **(A)** Mean classification accuracy (left) and standard deviation across 50 runs (right) for ConnCNN under five firing-rate modulation regimes. Presynaptic firing rates varied across successive temporal periods within the same simulation while preserving identical circuit connectivity and synaptic parameters. Four modulation regimes preserved the same overall mean firing rate across the full simulation: Base (constant 20 Hz), Mild (15–20–25 Hz), Moderate (10–20–30 Hz), and Strong (5–15–25–35 Hz). An additional Mean-shifted regime (5–15–30–45 Hz) simultaneously increased both modulation strength and global firing-rate statistics. Each row represents a CNN trained on one modulation dataset and tested across all modulation conditions. **(B)** Mean regression loss (left) and standard deviation across 50 runs (right) for WeightCNN under the same modulation benchmarking framework. WeightCNN remained robust across neighboring modulation regimes but exhibited progressively larger loss as the modulation difference increased.

For each modulation regime, CNNs were trained and evaluated using the same benchmarking procedure described previously for the perturbation analyses – a CNN was trained on one modulation dataset, evaluated on held-out test data from the same modulation condition, and subsequently tested on datasets from all other modulation regimes to assess cross-condition generalization. This train-test-generalization process was repeated over fifty independent runs, and the mean accuracy and standard deviation across runs are shown in Fig 6. As shown in Fig 6A, ConnCNNs trained on the Base, Mild, Moderate, and Strong modulation datasets generalized robustly across one another, with accuracies remaining near ceiling across most cross-testing conditions despite progressively shifted CCG indicator distributions, but failed markedly when tested on the Mean-shifted modulation dataset, with accuracies collapsing toward chance level (~50%). Interestingly, the Mean-shifted-trained model partially generalized back to the lower-modulation regimes. Performance degradation was accompanied by increased variability across runs, suggesting unstable generalization under large activity-distribution shifts. These results indicate that the CNNs tolerate substantial temporal firing-rate fluctuations and that CNN robustness is more sensitive to large shifts in global activity statistics than to temporal firing-rate modulation itself. We next applied the same global firing-rate modulation framework to WeightCNN to evaluate the robustness of synaptic weight inference under temporally varying population activity (Fig 6B). Compared with ConnCNN, WeightCNN generalized well across the Base, Mild, Moderate, and Strong modulation regimes, but showed progressively greater performance degradation as the disparity in modulation strength increased. In particular, models trained on weaker modulation regimes exhibited elevated MSE when tested on the Strong modulation condition, whereas the Strong-trained model partially generalized back to weaker regimes with intermediate performance degradation. Overall, these results further support the conclusion that synaptic weight inference is more sensitive to activity-distribution shifts than binary connectivity classification, and that exposure to broader training regimes promotes learning transfer.

### Cross-domain generalization to novel simulation and *in vitro* data

**Fig 7:**
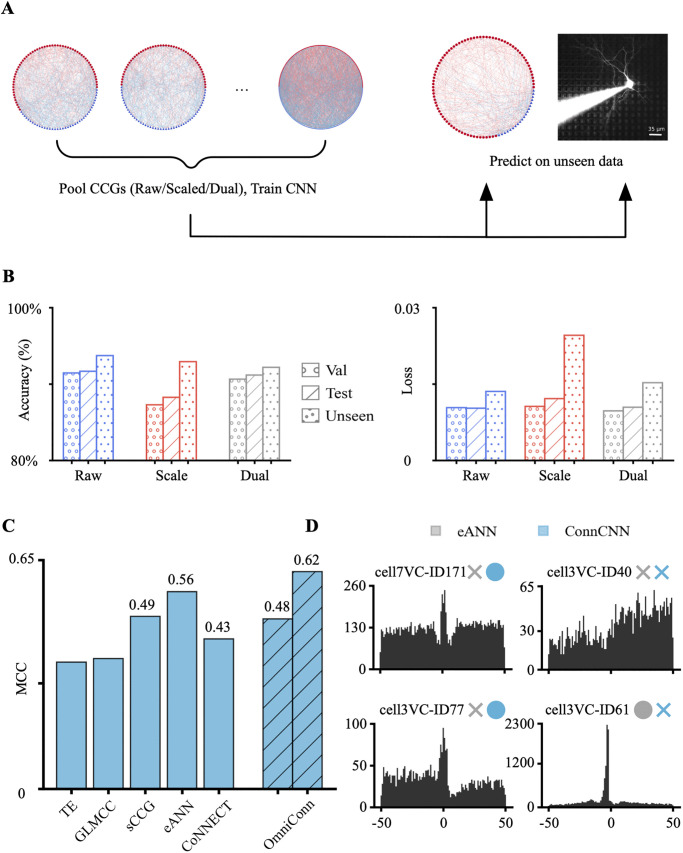
Cross-domain generalization to novel simulated and experimental datasets. **(A)** Schematic of the cross-domain evaluation pipeline. CNNs were trained on pooled CCGs (raw/scaled/dual inputs) from four simulated networks differing in topology and dynamics and then tested on CCGs from an unseen network and an *in vitro* HD-MEA dataset with patch-clamp-verified ground truth (data from Donner, et al. [23]). The rightmost HD-MEA/patch-clamp image panel was adapted from Donner, et al. [23]. **(B)** Model performance on unseen simulated network. Left: ConnCNN classification accuracy for raw-, scaled- and dual-input models. Right: corresponding WeightCNN regression performance (mean squared error, MSE). **(C)** Comparison of ConnCNN performance on the HD-MEA dataset with established methods, evaluated by MCC. Performance values for TE, GLMCC, sCCG, and eANN were reported in Donner et al. (2024) and are presented here for comparison. The MCC for CoNNECT was computed in this study by applying the publicly available pretrained model to the same HD-MEA dataset. OmniConn results correspond to models trained on four and six simulated networks, respectively, and evaluated on the same dataset. **(D)** Representative CCG examples illustrating prediction outcomes across models. Shown are cases where both models are incorrect, or where predictions differ between OmniConn and eANN. These examples highlight typical success and failure modes, including cases with unusually large CCG peaks that lead to misclassification.

To assess whether the cross-domain models OmniConn and OmniSyn can generalize beyond their training regime, we next evaluated in a more complex environment, including simulated recurrent networks and an *in vitro* HD-MEA dataset with a patch-clamp-verified ground-truth dataset [23] (**see Methods**). As illustrated in Fig 7A, we simulated multiple networks with different topologies and dynamics **(see** S3 Table, S8-S10 Figs), trained OmniConn and OmniSyn on pooled CCGs across networks, tested on CCGs from an unseen network and the *in vitro* HD-MEA dataset. When trained on a single network and tested on the unseen one, CNNs achieved accuracies spanning a large range: 71.20% to 93.99% for models trained on raw CCGs, 76.89% to 88.65% for models trained on scaled CCGs, and 74.77% to 93.41% for models trained on dual-input, and similarly for weight inference MSE loss: 0.0145 to 0.1665 (Raw), 0.0195 to 0.3839 (Scaled), and 0.0182 to 0.25 (Dual) (S11A-S11B Fig). The broad variability observed with raw CCG training indicates that some source networks generalized poorly to the unseen network, whereas models trained on scaled CCGs showed more stable but lower performance, consistent with the benchmarking analysis of scaled datasets. In contrast, dual-input models achieved both a narrower and higher accuracy range, combining the stability of scaling with the discriminative power of raw amplitude features. Expanding the training domain to four distinct simulated networks markedly improved OmniConn robustness (Fig 7B, **left**), yielding accuracies of 93.72% (Raw), 92.92% (Scaled), and 92.19% (Dual) on the unseen network dataset, even though the accuracies on the pooled testing and validation dataset were 2 – 5% lower. The corresponding OmniSyn (Fig 7B**, right**) achieved MSE of 0.0135 (Raw), 0.0246 (Scaled) and 0.0152 (Dual), indicating stable regression performance across heterogeneous network architectures. We further refined the models by training them on pooled CCGs from six networks after which the performance on the unseen network further improved: for connectivity prediction accuracy, we observed 95.2% (Raw), 92.4% (Scaled) and 94.1% (Dual); for weight inference loss, we observed an MSE of 0.0142 (Raw), 0.083 (Scaled) and 0.057 (Dual). Although OmniConn benefited from exposure to broader network diversity, OmniSyn showed a modest increase in MSE under scaled or dual-input conditions. This may indicate that, unlike discrete connectivity classification, continuous weight inference is more sensitive to inter-network variability in signal scale and dynamic range, resulting in slightly higher but more consistent loss values. These results support the notion that exposure to diverse network dynamics enables the OmniConn and OmniSyn to learn invariant representations of CCG structure that generalize across circuit topologies.

Next, we challenged the four-network and six-network OmniConn on the *in vitro* HD-MEA data, which contains 131 candidate connections, 26 of which were verified by paired patch-clamp recordings. As shown in Fig 7C, our model got MCC = 0.483 (four-network) and 0.62 (six-network model), compared with 0.56 for eANN and 0.43 for CoNNECT. Performance values, including MCC and true positive counts, for TE, GLMCC, sCCG and eANN were taken from Donner, et al. [23], while CoNNECT results were computed in this study using a publicly available pretrained model (**see Methods for more details**). For the experimentally confirmed subset, the four-network OmniConn correctly identified 19/26 true positives (recall = 0.73) and 80/105 true negatives (specificity = 0.76), corresponding to 25 false positives and 7 false negatives. The six-network OmniConn identified 16/26 true positives (recall = 0.62) and 100/105 true negatives (specificity = 0.95), corresponding to only 5 false positives but 10 false negatives. In comparison, eANN achieved 17/26 true positives (recall = 0.65) and 96/105 true negatives (specificity = 0.91; 9 false positives), while CoNNECT identified 13/26 true positives (recall = 0.50) and 89/105 true negatives (specificity = 0.85; 16 false positives). These results reveal a clear trade-off between sensitivity and specificity across methods. The four-network model achieves higher sensitivity (more true positives) but at the cost of increased false positives, whereas the six-network model substantially reduces false positives and achieves higher overall MCC, but at the expense of missing more true connections. This behavior highlights that the improved MCC of the six-network model is primarily driven by stronger control of false positives. Fig 7D presents representative CCG examples illustrating agreement and disagreement between models. One example missed connection (cell3VC-ID61) exhibited an unusually large CCG peak (~2300, compared with typically 60–1200), which likely saturated feature-map activations and inverted the learned response polarity, leading to a false negative. Together, these results indicate that a CNN trained solely on biologically diverse simulations can transfer to experimental recording without dataset-specific tuning. This cross-domain generalization suggests a promising path toward developing broadly applicable foundation models for neural connectivity inference capable of supporting large-scale, data-driven neuroscience in the future.

## Discussion

This study presents a systematic benchmarking and diagnostic investigation of convolutional neural networks (CNNs) for inferring monosynaptic connectivity and synaptic weight from spike-train cross-correlograms (CCGs). Through controlled perturbations of biophysical parameters and large-scale simulation sweeps, we dissect how CNN performance, internal representations, and generalization depend on network conditions, input scaling and training diversity. Our results reveal distinct representational regimes for connectivity classification versus weight regression, demonstrate how amplitude normalization reshapes signal sensitivity, and establish pooled CNNs trained on heterogeneous simulations as robust, transferable models that generalize to experimental data.

CNNs trained on homogeneous baseline datasets showed variable generalization across perturbations, often failing when biophysical statistics deviated from their training domain. For connectivity inference (ConnCNNs), models that generalized well exhibited bipolar feature map distributions and balanced reliance on global CCG structures. In contrast, models with unimodal activations tended to depend excessively on absolute amplitude cues and failed under parameter shifts, indicating overfitting to local signal strength. For synaptic-weight regression (WeightCNNs), no consistent bipolar pattern emerged even in robust models. Instead, WeightCNNs integrate multiple CCG cues in a less hierarchical manner, consistent with the observation that correctly and incorrectly inferred samples were intermixed in feature-indicator space. These results highlight that connectivity and weight inference engage distinct representational regimes, with the former anchored in a global temporal organization and the latter shaped by complex combinations of local and global features. These differences introduce competing requirements, as representations that are invariant to amplitude benefit classification, while preserving magnitude is critical for regression. As a result, unified models that attempt to infer both connectivity and weight may face inherent trade-offs. This may partly explain the reduced performance of the joint approaches such as CoNNECT in our evaluation. While unified architectures remain an interesting direction for further work, separating the two tasks allows each model to specialize in distinct aspects of the signal, leading to improved robustness and interpretability.

Training CNNs on pooled datasets that combined baseline conditions markedly improved cross-domain generalization. Both pooled OmniConn and OmniSyn maintained consistent performance across diverse biophysical perturbations and avoided catastrophic failures observed in single-domain models. This improvement stems from exposure to broader biophysical variability during training, which regularizes the learned feature space and discourages specialization to specific amplitude or timescale statistics [25,26]. Extending this strategy to multi-network training—pooling CCGs from several simulated networks with distinct topologies—further enhanced cross-domain robustness, enabling the model to generalize effectively to unseen simulated network architectures and even to *in vitro* HD-MEA recordings with patch-clamp-verified ground truth. Together, these results demonstrate that biologically grounded diversity, whether introduced through parameter perturbations or network heterogeneity, is key to building transferable CNN models for experimental neural data.

Amplitude normalization mitigated overfitting to CCG magnitude and improved robustness within single-parameter benchmarking perturbations. However, this benefit did not extend to pooled or cross-domain scenarios, where normalization attenuated informative amplitude features and degraded generalization. Absolute CCG amplitude reflects both nuisance variability and biophysically meaningful cues related to synaptic efficacy [16], but full normalization can suppress task-relevant information, particularly for weight inference. This trade-off illustrates a broader tension between variance reduction and the preservation of mechanistic signal features in data-driven neural models [27].

Importantly, this study fills a conceptual gap that has persisted despite decades of work on spike-based connectivity inference. Many of the principles revealed here, such as the need for training diversity, the dangers of amplitude-dominated representations, or the distinct failure modes of connectivity versus weight inference, may appear intuitive in hindsight. Yet the field lacked the diagnostic tools necessary to expose these effects. Prior work [18] primarily reported performance metrics from fixed datasets without mechanistic analyses of CNN representations, perturbation sensitivity, or domain shifts. As a result, generalization failures were often attributed to biological noise, whereas the potential role of representational biases intrinsic to CNNs has received comparatively less attention [18,28]. By combining controlled biophysical perturbations, interpretable feature analyses, and multi-domain pooled training, we reveal why CNNs fail, when they fail, and how these failures can be prevented. The framework introduced here thus converts what has traditionally been a black-box machine-learning problem into a mechanistically interpretable and principled approach to reliable circuit reconstruction.

An alternative approach to connectivity inference is to operate directly on raw spike timing data rather than cross-correlograms. Spike trains provide a richer and less processed representation of neuronal activity, and have been used in prior work to infer functional or monosynaptic connectivity, including both model-based and learning-based approaches [29–31]. However, their direct use introduces several challenges to CNN architectures. First, synaptic interactions are defined by precise temporal relationships between spikes across neurons, whereas standard convolutions operate on local patterns within each input channel and do not explicitly encode cross-neuron time-lag dependencies. As a result, a CNN applied to raw spike trains must implicitly learn cross-correlation structure from sparse binary signals, which is considerably more difficult than operating on CCGs where these relationships are explicitly represented. Second, spike trains are extremely sparse, making it challenging for convolutional filters to extract stable and informative features. Third, the representation of pairwise spike trains needs to be carefully designed, e.g., stacking versus concatenation, and different input organizations may lead to different inductive biases and learning dynamics. Nevertheless, direct spike-based modeling remains a promising direction, particularly for capturing higher-order interactions and global network structure. Future work may explore architectures that operate on population-level spike data, such as attention-based models, to integrate both local temporal structure and global dynamics in a unified framework.

Several limitations warrant consideration when interpreting the presented results. First, real-data validation was limited to a single *in vitro* dataset. Further work should extend these analyses to additional *in vitro* datasets exhibiting diverse firing statistics and, ultimately, to *in vivo* spike-train recordings to assess performance under more naturalistic conditions.

Second, synaptic connectivity in this study is inferred from cross-correlograms computed between individual neuron pairs, framing connectivity inference as a local, pairwise problem. While this approach is practical and experimentally relevant under current recording constraints, it is inherently limited by its restricted field of view: correlations observed between two neurons may arise not only from direct synaptic interactions but also from shared inputs, network-level dynamics, or global state fluctuations. Moreover, Volgushev, et al. [32] demonstrated that connection detectability and strength estimation improve as a larger fraction of presynaptic neurons are modeled within a GLM framework, highlighting the detrimental effects of subsampling on functional connectivity reconstruction. Unlike such multi-input models, the CNNs analyzed in this work operate on individual one-dimensional CCGs and therefore lack explicit contextual information about network structure or population activity. Although subsampling has been shown to affect network reconstruction accuracy in other frameworks [32,33], its specific impact on CNN-based inference remains unclear. Similar challenges were encountered historically in protein structure prediction, where early methods relied on pairwise residue statistics and coevolution signals but struggled to disambiguate direct physical contacts from indirect correlations mediated by other residues [34,35]. The success of AlphaFold 2 [36] highlighted the importance of jointly modeling all pairwise relationships under global consistency constraints, rather than treating pairwise interactions independently. Existing model-based approaches [37,38] that incorporate population activity partially address this issue by accounting for interactions among all recorded neurons, but they rely on predefined functional forms and do not learn global representations in a data-driven manner. Consequently, such methods do not fully resolve confounding effects arising from unobserved neurons or unknown network structure. Together, these considerations suggest that while local CNN-based inference provides a necessary and tractable solution at present, future work may benefit from incorporating broader population-level context and learned global representations, inspired by advances in structured deep learning models, to move toward more globally consistent connectivity inference.

Third, an important limitation concerns the dependence of CCG-based inference on the underlying dynamical state of the spiking network. While our perturbation framework systematically varies biophysical parameters, it does not explicitly control for or characterize distinct dynamical regimes of network activity, e.g., asynchronous irregular, synchronous, or oscillatory states. Prior work has shown that network state can strongly shape pairwise correlation and thus impact connectivity inference [39,40]. In E–I balanced networks operating in the asynchronous irregular regime, pairwise correlations can be substantially reduced despite the presence of direct synaptic connections, which may lower the signal-to-noise ratio of synaptic signatures in CCGs, thereby making connectivity inference more challenging [39,40]. In strongly recurrent networks, global dynamics can induce correlations between unconnected neurons, leading to systematic false positives that persist even with large amounts of data [14]. Since our current framework does not probe transitions between such regimes, the generalization of CNN-based inference across qualitatively different network states remains an open question. Future work should therefore incorporate control and characterization of network dynamics to better understand how state-dependent activity patterns influence connectivity and synaptic weight inference.

Fourth, the models examined here were restricted to conventional convolutional neural network (CNNs). In related domains such as image recognition, architectures incorporating residual connections (e.g., ResNet) have outperformed standard CNNs by mitigating vanishing-gradient issues and enabling deeper, more expressive feature hierarchies [41,42]. Incorporating similar architectural innovations may enhance inductive biases and improve generalization in connectivity or synaptic-strength inference tasks.

Finally, another important limitation concerns temporal subsampling. The current framework operates on spike-train-derived CCGs with millisecond resolution, whereas many experimental datasets—particularly those from calcium imaging—offer signals that are temporally downsampled and indirectly related to spiking activity. Applying CNN-based inference to calcium traces or dF/F-derived CCGs introduces additional temporal blurring and nonlinearities that can obscure precise synaptic signatures. Future work should therefore examine whether dedicated architectures and temporal encoding schemes can effectively capture and recover meaningful connectivity information under temporally subsampled conditions.

In summary, this work establishes a benchmark framework for evaluating and improving machine-learning models of synaptic inference. By linking performance degradation to concrete biophysical perturbations and internal feature structures, we identify the signal features and inductive biases that enable reliable generalization. Our findings highlight the importance of biologically grounded diversity and mechanistic interpretability as dual pillars for achieving robust, transferable models—advancing the development of practical, generalizable tools for large-scale neural circuit reconstruction.

## Methods and materials

### *In vitro* HD-MEA/patch-clamp dataset

#### Dataset description.

We used a publicly available dataset combining high-density microelectrode array (HD-MEA) recordings with simultaneous patch-clamp electrophysiology, originally reported in Donner, et al. [23]. The dataset consists of primary cortical neuron cultures prepared from embryonic day 18–19 Wistar rats and maintained *in vitro*. Recordings were performed at days *in vitro* (DIV) 17–18, when neuronal networks exhibit mature spontaneous activity. Extracellular activity was recorded using CMOS-based HD-MEAs comprising up to 26,400 electrodes, with simultaneous readout from up to 1024 channels at sampling rates of 10–20 kHz. In parallel, intracellular patch-clamp recordings were obtained from individual neurons under voltage-clamp conditions, enabling direct measurement of postsynaptic currents (PSCs) and providing ground-truth synaptic connectivity. Spike sorting of extracellular recordings was performed using Kilosort2 followed by manual curation, and only units meeting standard quality criteria (e.g., low refractory violations and sufficient spike counts) were retained. Connectivity labels were derived by linking extracellular spike trains of presynaptic neurons to intracellularly recorded PSCs using a regression-based framework, allowing identification of monosynaptic connections. This dataset provides simultaneous population-level spiking activity and ground-truth synaptic connectivity, enabling quantitative evaluation of inference methods on biologically realistic data.

#### Spike-train feature extraction.

Neuronal activity statistics (S9 Fig) were computed directly from the raw spike-time data provided by Donner, et al. [23] (NPZ format). For each recording, spike times and corresponding neuron identities were used to reconstruct per-neuron spike trains within a defined analysis window. For each neuron, three activity features were computed. The firing rate was defined as the total number of spikes divided by the global recording duration. Inter-spike interval variability was quantified using the coefficient of variation (ISI CV), computed as the ratio of the standard deviation to the mean of successive inter-spike intervals. To capture slower temporal variability in firing activity, the Fano factor was calculated as the variance-to-mean ratio of spike counts across non-overlapping temporal bins (bin size = 100 ms). To ensure consistency with the connectivity inference analysis, we restricted the analysis to the subset of neurons associated with the curated CCGs (S12 Fig) provided by Donner, et al. [23].

### Neural simulation framework

We implemented a hierarchical simulation framework consisting of two levels: a minimal 40→1 circuit used for benchmarking analyses and a larger recurrent network used to model diverse circuit dynamics. Both were built from the same biophysical components described below, including stochastic presynaptic spike generation, background noise input, and a conductance-based leaky integrate-and-fire (LIF) neuron model.

#### Poisson spike train generation and bursting activity.

To increase the biological realism and challenge the robustness of connectivity inference, we incorporated burst spiking into presynaptic spike trains. Presynaptic spike trains were generated as homogeneous Poisson processes, with additional bursting activity introduced through a discrete geometric-distribution process [15]: bursting was incorporated by probabilistically inserting short inter-spike intervals (ISIs) following each spike. For each spike occurring at index i, a random ISI was drawn from a uniform distribution


$$\begin{array}{c} \Delta t\;\sim \;Uniform(a,\;b) \end{array}$$

where a=2 ms and b=7 ms. A subsequent spike was added at time $t_{i}+\Delta t$. The burst continued probabilistically according to a geometric process: after each additional spike, a random number u~Uniform (0, 1) was drawn, and the burst was extended while u>p, where p is the burst-termination probability (BP). Consequently, the number of spikes K in a burst (including the initiating spike) follows


$$\begin{array}{c} K\sim Geom(p),Pr\;[K=k]\;=(\text{1}-p{)}^{k-\text{1}}p,\;k=\text{1},\text{2},\ldots \end{array}$$

A larger p produces shorter bursts (e.g., p=0.5 yields bursts of ≈2 spikes on average), while smaller p yields longer bursts (e.g., p=0.2 yields ≈5 spikes per burst). When p=1.0, no additional spikes are added. Each added spike is placed at discrete time index i+Δt/dt if it falls within the simulation window. The procedure preserves the total duration of the spike train while increasing its local spike density around burst initiations, thereby emulating biologically realistic bursting firing with stochastic burst lengths. To prevent unrealistically short ISIs, all spike trains were post-processed to enforce an absolute refractory period (APR) of 2 ms.

#### Background pink noise.

To model spontaneous synaptic drive, the postsynaptic neuron received a stochastic current $I_{noise}(t)$ governed by an Ornstein-Uhlenbeck (OU) process [23,43]. This process produces noise with exponentially decaying temporal correlations:


$$\begin{array}{c} \tau_{noise}\frac{dI_{noise}(t)}{dt}=\;-I_{noise}(t)+\;\sigma_{noise}\sqrt{\text{2}\tau_{noise}dt}\xi (t), \end{array}$$

where $\tau_{noise}$ is the time constant controlling the smoothness of fluctuations, $\sigma_{noise}$ is the noise amplitude and ξ(t) is Gaussian white noise $\xi_{t}\sim N(\text{0},\;\text{1})$ at each timestep. In discrete form (Euler-Maruyama integration, step dt):


$$\begin{array}{c} I_{t+\Delta t}=I_{t}+\Delta t\left(-\frac{I_{t}}{\tau_{noise}}\right)+\sigma_{noise}\;\sqrt{\text{2}\frac{\Delta t}{\tau_{noise}}}\xi_{t},\;\xi_{t}\sim N\;(\text{0},\;\text{1}) \end{array}$$

The mean drive $\mu_{noise}$ is then added to shift the current’s operating point, yielding


$$\begin{array}{c} I_{noise}(t)=\;\mu_{noise}+\tilde{I}(t), \end{array}$$

where $\tilde{I}(t)$ is the zero-mean OU fluctuation. Smaller $\tau_{noise}$ values generate rapidly varying inputs, whereas larger values yield slowly drifting fluctuations.

#### Leaky integrate-and-fire model.

A conductance-based leaky integrate-and-fire (LIF) model [43,44] was adopted to simulate the membrane potential dynamics and spiking activity of individual neurons obeying:


$$\begin{array}{c} \tau_{m}\frac{dV(t)}{dt}=\;-\left(V(t)-V_{rest}\right)\;-\frac{g_{syn}^{E}(t)}{g_{L}}\left(V(t)-E_{E}\right)-\;\frac{g_{syn}^{I}(t)}{g_{L}}\left(V(t)-E_{I}\right)+\frac{I_{noise}(t)}{g_{L}} \end{array}$$

$V_{rest},\;E_{E},\;E_{I}$ denote leak, excitatory and inhibitory reversal potentials, set to −70 mV, 0 mV and −75 mV, respectively. A spike was emitted when $V\;\geq V_{thresh}=-\text{5}\text{5}\;mV$, after which the membrane potential is reset to $V_{reset}=-\text{7}\text{0}\;mV$, and held in an absolute refractory period (APR) $\tau_{ref}=\text{2}\;ms$. The leak conductance was fixed at $g_{L}=\text{1}\text{0}\;nS$, corresponding to a membrane capacitance of $C_{m}=\tau_{m}g_{L}=\text{1}\text{5}\text{0}\;pF$. The conductance transient change in the postsynaptic neuron j’s conductance $g_{syn}$ produced by presynaptic neuron i’s spiking activity follows a simple ordinary differential equation:


$$\begin{array}{c} \frac{dg_{syn}^{i\to j}(t)}{dt}=-\frac{g_{syn}^{i\to j}(t)}{\tau_{syn}}+\;{\overset{―}{g}}_{syn}w_{i\to j}\sum_{f}\delta (t-t_{i}^{\left(f\right)}) \end{array}$$

where $\tau_{syn}$ is the synaptic time constant, ${\overset{―}{g}}_{syn}$ is the maximum conductance elicited by each incoming spike, and $w_{i\to j}$ is the synaptic weight from neuron i to neuron j, and $t_{i}^{\left(f\right)}$ denotes the f-th spike time of neuron i. Each presynaptic spike instantaneously increases $g_{syn}^{i\to j}\;$by ${\overset{―}{g}}_{syn}w_{i\to j}$, after which the conductance decays exponentially with time constant $\tau_{syn}$.

#### Minimal circuit simulation.

To benchmark CNN generalization under biophysically variable parameters, a minimal 40→1 circuit was simulated, comprising 40 presynaptic neurons (excitatory + inhibitory) projecting to one postsynaptic excitatory neuron. Each simulation ran for 600 s (600,000 ms) with a 1 ms resolution. Presynaptic neurons were configured as follows:

i) The firing rates were set to 20 Hz for excitatory neurons and 25 Hz for inhibitory neurons;
ii) Bursting levels were adjusted according to the benchmarking design (S1 Table);
iii) Synaptic parameters were fixed at ${\overset{―}{g}}_{E\to E}=\text{1}.\text{0}\;nS$, ${\overset{―}{g}}_{I\to E}=\text{0}.\text{7}\;nS$, $\tau_{E}=\text{5}\;ms,\;$and $\tau_{I}=\text{1}\text{0}\;ms$. Synaptic weights $w_{i\to j}$ were systematically varied to test sensitivity, with $w_{min}=\text{0}.\text{4}$ as the smaller effective values that produced reliable postsynaptic firing and measurable CCGs;
iv) The excitatory/inhibitory ratio was varied across configurations, excluding cases with E/I < 0.3.

For the postsynaptic neuron:

i) The leak conductance and membrane parameters were set to $g_{L}=\text{1}\text{0}\;nS,\;\tau_{m}=\text{1}\text{5}\;ms,\;V_{init}=-\text{6}\text{5}\;mV$;
ii) Noise input parameters were fixed $\tau_{noise}=\text{1}\text{5}$ and $\sigma_{noise}=\text{5}$;
iii) The mean drive $\mu_{noise}$ was systematically varied to examine how background input strength modulates the detectability of pre-post connectivity.

#### Network simulation.

We simulated seven LIF networks spanning different operating regimes to mimic diverse circuit states (e.g., active vs. inactive, large vs. small, dense vs. sparse). Here we describe one representative network, whereas population-level schematics of all simulated circuits are provided in S8 Fig following the graphical notation proposed by Senk, et al. [45], and detailed parameters for the remaining six are provided in S3 Table. Each simulation ran for 600 s (600,000 ms) with a 1 ms time step. The network comprised 20 external neurons (10 excitatory $X_{E}$ and 10 inhibitory$\;X_{I}$) that provided stochastic external drive to 100 recurrent LIF neurons (80 excitatory $S_{E}$ and 20 inhibitory $S_{I}$). External spike trains were generated following Poisson processes with added bursting activity (burst-termination probability was set BP ~ Uniform(0.8, 1.0) for $X_{E}$ and BP ~ Uniform(0.6, 0.8) for $X_{I})$ and were post-processed by an ARP=2ms. Mean firing rates were drawn from FR ~ Uniform(20, 30) for $X_{E}$ and FR ~ Uniform(25, 30) for $X_{I}$. To keep total synaptic drive comparable across network sizes, the base conductance was normalized as $\overset{―}{g}=\text{2}.\text{5}\;nS/\sqrt{S_{E}+S_{I}}$ following standard balanced-network scaling principles [44,46]. External neurons were randomly connected to recurrent population with probability 0.1, using maximum conductance $\overset{―}{g}$ and connection weights $W_{X_{E}\to S_{E}},\;W_{X_{E}\to S_{I}}\;\sim \;Uniform\;(\text{1}.\text{0},\;\text{1}.\text{5})$ weights from $W_{X_{I}\to S_{E}},\;W_{X_{I}\to S_{I}}\;\sim \;Uniform\;(\text{0}.\text{5},\;\text{1}.\text{1})$. For the recurrent neurons, excitatory cells $S_{E}$ had time constant, leak channel conductance and excitatory synapse time constant $\tau_{m}^{E}=\text{1}\text{5}\;ms,\;g_{L}^{E}=\text{1}\text{0}\;nS,\;\tau_{syn}^{E}=\text{3}\;ms$ and inhibitory cells had $\tau_{m}^{I}=\text{8}\;ms,\;g_{L}^{I}=\text{1}\text{5}\;nS,\;\tau_{syn}^{I}=\text{5}.\text{7}\;ms$, consistent with typical cortical excitatory and inhibitory neuron biophysics [46–49]. To introduce intrinsic heterogeneity, the initial membrane potentials of recurrent neurons were drawn from a truncated Gaussian distribution centered at −65 mV (μ=−65, σ=2, bounded between −70 mV and −60 mV). Each neuron also received OU background input: excitatory neurons had $\mu_{E}=\text{1}\text{8}\text{0}\;pA$ with $\tau_{noise}^{E}\sim Uniform\;(\text{1}\text{5},\;\text{3}\text{0})ms\;$and $\sigma_{noise}^{E}\sim Uniform(\text{3},\;\text{5})$, inhibitory neurons had $\mu_{I}=\text{2}\text{0}\text{0}\;pA$ with $\tau_{noise}^{I}\sim Uniform\;(\text{2}\text{5},\;\text{3}\text{0})ms\;$and $\sigma_{noise}^{I}\sim Uniform(\text{3},\;\text{5})$. Synaptic weight between neurons was defined as $g_{i\to j}=\;{\overset{―}{g}}_{syn}w_{i\to j}$. Class-specific conductance scales were implemented as:


$$\begin{array}{c} {\overset{―}{g}}_{E\to E}=\;\alpha_{E\to E}\overset{―}{g},\;{\;\overset{―}{g}}_{E\to I}=\;\alpha_{E\to I}\overset{―}{g},\;{\;\overset{―}{g}}_{I\to E}=\;\alpha_{I\to E}\overset{―}{g},\;{\;\overset{―}{g}}_{I\to I}=\;\alpha_{I\to I}\overset{―}{g} \end{array}$$

With multipliers $\alpha_{E\to E}=\text{1}.\text{0},\;\alpha_{E\to I}=\text{3}.\text{5},\;\alpha_{I\to E}=\text{2}.\text{0},\;$and $\alpha_{I\to I}=\text{1}.\text{5}$, ensuring stronger inhibitory pathways to maintain E-I balance. Corresponding weight distributions were: $w_{E\to E}\;\sim \;Uniform(\text{1}.\text{0},\;\text{1}.\text{5}),\;w_{E\to I}\;\sim \;Uniform(\text{1}.\text{5},\;\text{2}.\text{5}),\;w_{I\to E}\;\sim \;Uniform(\text{1}.\text{0},\;\text{1}.\text{5}),$ and $w_{I\to I}\;\sim \;Uniform(\text{0}.\text{5},\;\text{1}.\text{0})$. Recurrent connectivity was sparse, with connection probabilities of 0.1 among same-type neurons ($S_{E}\to S_{E},\;S_{I}\to S_{I}$) and denser cross-type connectivity ($S_{E}\to S_{I},\;S_{I}\to S_{E}$) at 0.25. These parameters jointly produced a dynamically balanced E-I network that exhibited stable, irregular spiking activity (S9-S10 Figs).

### Design, optimization, and training of Convolutional Neural Networks

Convolutional neural networks (CNNs) provide a natural inductive bias for inferring synaptic connectivity from CCGs, which are one-dimensional temporal signals characterized by localized, structured deflections around zero time lag. Synaptic effects manifest as short-latency peaks or troughs spanning only a few milliseconds, while background correlations and noise vary more slowly over time. By hierarchically combining local features across neighboring time bins, CNNs can integrate fine-scale synaptic signatures with broader contextual structure in the CCG, making them effective for distinguishing direct monosynaptic interactions from background activity fluctuations.

#### Design choice for separate connectivity and weight inference models.

We treat connectivity classification and synaptic weight estimation as two distinct tasks in this study. In practice, weight estimation requires resolving fine-scale amplitude differences in the CCG, while connectivity classification primarily depends on detecting the presence of structured deviations from background activity [16]. These tasks impose different requirements on feature representation and preprocessing. Accordingly, we train separate CNN models for connectivity classification and weight regression, allowing each network to specialize in different aspects of the CCG signal.

#### Convolutional neural network architecture.

The synthetic dataset of one simulated network was used as a controlled benchmark for optimizing the CNN architecture, including number of convolutional layers, filter sizes, activation functions and learning rate. We initially adopted a structure similar to that of Endo, et al. [18] but this configuration exhibited substantial overfitting. We progressively simplified the architecture by reducing the number of convolutional layers from three to two, and systematically explored a range of filter sizes (10−80) and activation functions (Tanh and ReLU). The final model comprised two convolutional blocks followed by global feature aggregation and a fully connected output layer. The first convolutional layer (1→4 channels, kernel size = 70, stride = 1) captures broad temporal motifs across approximately 70 ms of the CCG signal, followed by batch normalization and a tanh nonlinearity. The second convolutional block (4→2 channels, kernel size = 35) refines mid-range temporal patterns, again followed by a batch normalization and tanh activation. The resulting feature maps are reduced using an adaptive global average pooling layer, which collapses each channel to a single scalar value, producing a two-dimensional representation sample. A dropout layer (p=0.3) provides regularization before a fully connected linear layer (2→1) generates the output logit. This scalar output represents the predicted connection probability or synaptic weight between the neuron pair. The total number of trainable parameters is approximately 600, allowing the model to capture essential CCG dynamics while maintaining interpretability and avoiding overfitting.

We implemented a dual-input variant that processes the raw and amplitude-normalized CCGs in two parallel, independently parameterized 1D-CNN branches (identical architecture, no weight sharing). Each branch comprises two conv–BN–Tanh blocks as described above, followed by adaptive global average pooling to a 2-dimensional feature vector. The two pooled vectors are concatenated and passed through dropout layer and a shared fully connected layer (4→1) produces the output logit. Using separate filters per branch allows the network to learn distinct feature detectors tailored to the very different magnitude ranges of raw versus scaled CCGs, while retaining a compact parameter budget (≈1.2k).

#### Training sample generation.

In the minimal 40→1 benchmarking circuit, positive samples (presence of monosynaptic connection between a neuron pair) were defined as CCGs computed between each of the 40 presynaptic neurons and the single postsynaptic neuron, yielding 40 connected CCG samples per simulation. Negative samples (absence of monosynaptic connection) were generated to match this count by, for each presynaptic neuron, randomly selecting one neuron from the remaining 39 presynaptic neurons and computing the CCG between the selected pair. This procedure produced 40 negative samples per simulation, ensured balanced class sizes and avoided introducing direct synaptic interactions in the negative class by construction. For recurrent network, CCG samples were generated from pairs of recurrent neurons ($S_{E},\;S_{I}$). Positive samples were defined as CCGs between neuron pairs with a direct synaptic connection according to the ground-truth connectivity matrix. For each recurrent neuron, negative samples were generated by pairing that neuron with other recurrent neurons to which it was not directly connected. To control class balance, the number of negative samples for each neuron was matched to the number of its positive samples by randomly selecting from the available unconnected neuron pairs. Samples were randomly shuffled and split into training, validation, and test sets using stratified sampling to preserve the proportion of connected and unconnected pairs across all splits (70% training, 15% validation, 15% test). All random operations were performed with fixed seeds to ensure reproducibility.

#### Task formulation and labeling strategy.

We formulated synaptic inference primarily as a binary classification task, predicting the presence or absence of a monosynaptic connection between a neuron pair. Although multi-class formulations that distinguish excitatory, inhibitory, and non-connected pairs have been proposed in prior work, we adopted a binary formulation for three reasons. First, empirical comparison showed that binary classification achieves performance comparable to, and in several cases more stable than, three-class classification across unseen simulated networks and experimental data (S13 Fig). In particular, the three-class model exhibited higher variance and reduced robustness, likely due to the strong imbalance and limited separability of inhibitory connections. Second, real experimental ground-truth dataset [23] provides only binary connectivity labels, without reliable excitatory or inhibitory annotations. Consequently, three-class predictions cannot be meaningfully validated on experimental data. Third, from a practical experimental perspective, determining synapse type (excitatory vs. inhibitory) typically requires additional intracellular recordings, pharmacological manipulations, or cell-type–specific labeling, whereas binary connectivity is more readily accessible. We therefore focus on binary connectivity inference as a task that is both experimentally grounded and robust to domain shift.

#### Model optimization and convergence evaluation.

Before final training, we conducted a systematic search to identify suitable learning rates and epoch lengths for each dataset (e.g., network-level dataset and 40→1 minimal circuits). For each dataset, combinations of learning rate (lr∈{0.001, 0.003, 0.005}) and training duration (E∈{100, 120, 150, 200, 250, 300} epochs) were tested in a grid-like fashion. Each combination was trained five times with independent random initializations to ensure stability. During these exploratory runs, we plotted training and validation loss trajectories ($L_{train},\;L_{val}$) for each configuration and examined their convergence profiles. The optimal epoch number and learning rate were selected as configuration showing i) stable convergence of $L_{train}$ showing not divergence between training and validation loss, and ii) minimal $L_{val}$ at a plateau.

#### Training procedures.

For finalized models to infer connectivity, training was performed using binary cross-entropy with logits loss (BCEWithLogitsLoss), optimized with the Adam algorithm ($lr=\text{0}.\text{0}\text{0}\text{3},\;\beta_{\text{1}}=\text{0}.\text{9},\;\beta_{\text{2}}=\text{0}.\text{9}\text{9}\text{9}$). The learning rate was adaptively reduced using a ReduceLRONPlateau scheduler (factor = 0.5, patience = 10 epochs), which halved the rate when validation loss failed to improve. Each training epoch involved forward and backward passes over all mini batches from the training loader, followed by evaluation on the validation set. During validation and testing, model predictions were thresholded at 0.51 to classify each neuron pair as connected (1) or unconnected (0). Performance metrics included average validation loss and classification accuracy. The best-performance epoch (minimum validation loss) was selected as the final model checkpoint.

For synaptic weight inference, the same CNN architecture was trained in a regression setting using a custom Huber loss function with adaptive weighting (HuberLossWithWeight). This criterion emphasized small-magnitude weights near zero to improve sensitivity around weak connections through an exponential weighting term


$$\begin{array}{c} w=\text{exp}\left(-\lambda \left|w_{i}^{true}\right|\right), \end{array}$$

with an additional enhancement when $\left|w_{i}^{true}\right|<\text{0}.\text{1}$, thereby increasing sensitivity to weak synapses. On top of this intrinsic weighting, the training loop further upweighted all non-zero synaptic weights by a factor of 1+α (α=5), yielding a batch loss


$$\begin{array}{c} L=\frac{\text{1}}{B}\sum_{i=\text{1}}^{B}\left(\text{1}+\alpha {\text{1}}_{\left\{w_{i}>\text{0}\right\}}\right)\ell_{i}, \end{array}$$

where $\ell_{i}$ is the per-sample Huber loss returned by the criterion, and B is the batch size. This dual-level weighting scheme enhances model’s ability to resolve weak-but-nonzero synapses while preventing the regression objective from being dominated by a large number of zero-weight pairs. Optimization was performed using the Adam algorithm ($lr=\text{0}.\text{0}\text{0}\text{5},\beta_{\text{1}}=\text{0}.\text{9},\;\beta_{\text{2}}=\text{0}.\text{9}\text{9}\text{9},$ weight decay = $\text{1}\times {\text{1}\text{0}}^{-\text{5}}$), paired with a cosine-annealing learning rate scheduler ($T_{max}=\text{1}\text{0},\;\eta_{min}={\text{1}\text{0}}^{-\text{6}})$. During evaluation, the Huber loss was computed separately for zero-weight and non-zero-weight synapses, providing detailed performance diagnostics across connection classes.

### Cross-correlogram computation

For each ordered neuron pair, we computed a raw cross-correlogram (CCG) from the spike times of the putative presynaptic and postsynaptic neurons. Spike times were represented in milliseconds. For each pair, all pairwise spike-time differences were computed as


$$\begin{array}{c} \Delta t=t_{post\;}-t_{pre}. \end{array}$$

These differences were binned into a histogram over a ±50 ms window using 1 ms bins. Positive lags therefore correspond to postsynaptic spikes occurring after presynaptic spikes. The same CCG computation procedure was applied to simulated spike trains and to spike trains from the *in vitro* HD-MEA dataset. The resulting raw CCG consisted of unnormalized spike-count histograms, which was used as input to raw-input CNN models.

For amplitude-normalized models, each CCG was scaled relative to the fluctuation level estimated from outer two tails of the CCG (25 bins from two tails were used). This scaling reduced dependence on absolute CCG magnitude while preserving the temporal structure of the peak or dip around zero lag. Dual-input models received both the raw CCG and the amplitude-normalized CCG as parallel inputs. This CCG construction is consistent with the raw spike-time-difference histogram used in GLMCC [16] and eANN [23] frameworks.

### CCG indicators

To quantify characteristic features of CCGs, we defined a set of analytical indicators that capture both shape properties (e.g., peak/dip amplitude) and information content (e.g., entropy, KL divergence). This procedure was designed to accommodate both excitatory (peak-shaped) and inhibitory (dip-shaped) interactions. Let the CCG be sampled in K=2N+1 bins with bin size Δt (ms). Index bins by k∈{0, …, 2N} and the center bin is c=N. Let $W_{f}\;(ms)$ be a symmetric focus window around the center and $W_{b}\;(ms)$ the background window length taken from the two tails.

Within the focus window $I_{f}=\left\{k:\;N-\left\lfloor \frac{W_{f}}{\Delta t}\right\rfloor \;\leq k\leq \;N+\left\lfloor \frac{W_{f}}{\Delta t}\right\rfloor \right\}$, we locate the most prominent deflection i, either maximum for excitatory (peak) or the minimum for inhibitory (dip) and denote the values as $x_{i}$, corresponding to the peak height or dip depth. A background activity level was estimated from the mean and standard deviation of two tails of the CCG (length $W_{b}/\Delta t$), denoted as $\mu_{baseline}\text{ and }\sigma_{baseline}$:


$$\begin{array}{c} B=\left\{\text{0},\;\ldots ,\;L_{b}-\text{1}\right\}\cup \left\{\text{2}N-L_{b}+\text{1},\;\ldots ,\;\text{2}N\right\},\;L_{b}=\;\left\lfloor \frac{W_{b}}{\Delta t}\right\rfloor \;, \end{array}$$

$$\begin{array}{c} \mu_{baseline}=\frac{\text{1}}{\left|ℬ\right|}\sum_{k\in B}x_{k},\;\sigma_{baseline}=\;\sqrt{\frac{\text{1}}{\left|B\right|-\text{1}}{\sum_{k\in B}{(x}_{k}-\mu_{baseline})}^{\text{2}}}\;. \end{array}$$

The peak/dip-to-noise ratio was computed as


$$\begin{array}{c} P\text{2}N=\left\{ \begin{array}{cc} \frac{x_{i}-\mu_{baseline}}{\sigma_{baseline}+\epsilon },\; & excitatory\\ \frac{\mu_{baseline}-x_{i}}{\sigma_{baseline}+\epsilon },\; & inhibitory \end{array}\right.\;with\;\varepsilon ={\text{1}\text{0}}^{-\text{1}\text{0}}). \end{array}$$

Temporal span was defined by thresholding the smoothed CCG around the deflection using a polarity-specific dynamic threshold:


$$\begin{array}{c} \theta =\left\{ \begin{array}{cc} \mu_{baseline}+\text{2}\sigma_{baseline}\;,\; & excitatory,\\ \mu_{baseline}-\text{2}\sigma_{baseline},\; & inhibitory, \end{array}\right. \end{array}$$

For information-theoretic measures, let ${\tilde{x}}_{k}$ be a smoothed version of $x_{k}$ (Gaussian kernel with std $\sigma_{ker}=\text{0}.\text{5}$). To obtain a probability mass function, ensure nonnegativity (shift by minimum):


$$\begin{array}{c} y_{k}=\;{\tilde{x}}_{k}-\;\underset{j}{\text{min}}{\tilde{x}}_{j}+\;\varepsilon \;,\;P_{k}=\frac{y_{k}}{\sum_{j=\text{0}}^{\text{2}N}y_{j}}\;. \end{array}$$

The normalized Shannon entropy was defined as


$$\begin{array}{c} H_{norm}=\;-\frac{\sum_{k=\text{0}}^{\text{2}N}P_{k}{\text{log}}_{\text{2}}P_{k}}{{\text{log}}_{\text{2}}\left(\text{2}N+\text{1}\right)}\;. \end{array}$$

Within the central $\pm W_{f}$ ms window (support size $K_{f}$), we also reported the KL divergence from a uniform distribution $U_{k}=\frac{\text{1}}{K_{f}}$:


$$\begin{array}{c} D_{KL}(P||U)=\;\sum_{k\in I_{f}}P_{k}{\text{log}}_{\text{2}}\left(\frac{P_{k}}{U_{k}}\right)={\text{log}}_{\text{2}}K_{f}-\;\sum_{k\in I_{f}}P_{k}{\text{log}}_{\text{2}}P_{k} \end{array}$$

### Model ranking by balanced perturbation performance

For each baseline CNN, we evaluated model performance separately on two disjoint subsets of the test set: i) self-perturbation subset, containing samples with varying values of the same parameter that was changed to build the baseline CNN; ii) other-perturbation subset, the remaining samples. For every sample, we defined a binary hit indicator as 1 if the predicted label matched the ground-truth connectivity label or MSE < 0.04 for regression task, else 0. For performance reference, we computed:


$$\begin{array}{c} h_{self}^{\left(b\right)}=\frac{\text{1}}{n_{self}^{\left(b\right)}}\sum hit,\;h_{other}^{\left(b\right)}=\frac{\text{1}}{n_{other}^{\left(b\right)}}\sum hit \end{array}$$

where $n_{self}^{\left(b\right)}\;$ and $n_{other}^{\left(b\right)}$ are the numbers of self/other samples, respectively. To obtain a single balanced score per model, we use the harmonic mean of the two hit rates, defined as


$$\begin{array}{c} H^{\left(b\right)}=\frac{\text{2}h_{self}^{\left(b\right)}h_{other}^{\left(b\right)}}{h_{self}^{\left(b\right)}+h_{other}^{\left(b\right)}}, \end{array}$$

which penalizes imbalance between the two subsets. We estimated 95% confidence intervals (CI) for $H^{\left(b\right)}$ via a parametric bootstrap (binomial resampling), i.e., for each model we drew B=2000 pairs


$$\begin{array}{c} {\tilde{h}}_{self}\sim Binom(n_{self}^{\left(b\right)},\;h_{self}^{\left(b\right)})/n_{self}^{\left(b\right)},\;{\tilde{h}}_{other}\sim Binom(n_{other}^{\left(b\right)},\;h_{other}^{\left(b\right)})/n_{other}^{\left(b\right)}, \end{array}$$

computed $\tilde{H}=\text{2}{\tilde{h}}_{self}{\tilde{h}}_{other}/({\tilde{h}}_{self}+\;{\tilde{h}}_{other})$, and took the 2.5^th^/97.5^th^ percentiles of $\tilde{H}$ as the CI. A fixed random seed (42) was used for reproducibility. Model ranking was performed primarily by $H^{\left(b\right)}$, ties were broken by $h_{self}^{\left(b\right)}$, then by $h_{other}^{\left(b\right)}$, and finally by the baseline name for stability.

### Diagnostic metrics: Feature-map mean, Random Forest classification, and SHAP analysis

To interpret CNN behavior, we analyzed the internal activations of the final convolutional layer. After the second convolutional block, each input CCG produced two feature maps, which were reduced by adaptive global average pooling. We refer to the resulting two scalar pooled activations as feature-map means (FMM1 and FMM2). These values summarize how strongly each learned convolutional channel responds to a given CCG and provide a low-dimensional representation of the model’s internal response.

To relate CNN performance to CCG signal properties, we trained a Random Forest classifier to predict whether each CCG input was correctly classified by the CNN using CCG statistics as input. The classifiers were implemented using RandomForestClassifier from *scikit-learn* [50] with default parameters.

To quantify the contribution of each indicator to prediction outcomes, we computed SHAP (Shapley Additive exPlanations) values using the TreeExplainer from the SHAP Python package [51] with default parameters. CCG indicators contributions were summarized as the mean absolute SHAP values across samples, and to enable comparison across all baseline CNN models, we normalized these values by dividing each feature’s value by the sum of mean absolute SHAP values across all features. These normalized values (relative SHAP) indicate the relative importance of each CCG indicator, with larger values reflecting stronger contributions to distinguishing correctly predicted from missed samples.

### Computational resources and implementation

All simulations, data processing, and analyses were implemented in Python using custom-written code. Spiking network simulations were performed using an in-house implementation of leaky integrate-and-fire (LIF) neurons to allow precise control over model parameters and perturbations. CCG computation, dataset construction, and diagnostic analyses were implemented using custom Python scripts, together with standard scientific computing libraries. CNN networks were implemented using PyTorch framework. Model training was performed on GPU hardware (NVIDIA Tesla V100-SXM2–16GB), while data preprocessing and analysis were conducted on CPU nodes with memory ranging from 8GB to 64GB depending on dataset size. The full computational pipeline has been organized into modular scripts and is available in the public GitHub repository (https://github.com/XiaoqianSun0104/OmniCNN_Infer_Connectivity). Instructions for environment setup and execution are provided to facilitate reproducibility.

### Comparison with existing methods on HD-MEA data

To assess performance on experimental data, we compared OmniConn against previously reported methods using the HD-MEA/patch-clamp dataset from Donner, et al. [23]. Performance metrics for TE, GLMCC, sCCG and eANN were taken directly from that study, where all methods were evaluated on the same dataset with identical ground-truth labels and evaluation metrics. For CoNNECT, which was not evaluated in Donner, et al. [23], we obtained the publicly available pretrained model from the author’s repository (https://github.com/shigerushinomoto/CoNNECT) and applied it to the same HD-MEA dataset used in this study. Importantly, all comparisons are based on the same experimental dataset, ground-truth connectivity labels, and CCG computation procedure.

## Supporting information

S1 FigDistributions of CCG indicators across baseline regimes.Histograms showing the distribution of key CCG indicators computed from baseline training datasets under low-end and high-end regimes. Each row compares one perturbation axis: μ150 vs. μ180 (background input), BP1.0 vs. BP0.5 (bursting probability), E3 vs. E8 (E/I ratio), and w0.5 vs. w1.5 (synaptic weight scaling). Columns correspond to peak height/dip depth, peak-to-noise/dip-to-noise ratio, normalized entropy, and KL divergence of the focused CCG window. Low-end regimes are shown in blue and high-end regimes in purple. (A) Histograms for excitatory samples. (B) Histograms for inhibitory samples.(TIF)

S2 Fig Feature map mean distribution of all baseline models. (A) Feature map mean (FMM) distributions for all baseline models in the connectivity-inference task on test data loader, left panel - excitatory samples and right panel - inhibitory samples. Models are colored by performance score balancing model performance in self- and cross-perturbation tests. (B) FMM distributions for all baseline models in weight inference task.
(TIF)

S3 FigSHAP feature attribution of all five indicators for connectivity inference model.SHAP attribution for connectivity inference task, top - excitatory, bottom - inhibitory. The most robust model across perturbations, μ180, showed distributed feature attribution across all five indicators, which showed a pattern similar to that of OmniConn models in the inhibitory scenario. The least robust model, μ165, relied heavily on peak height. Because OmniConn models produced too few failed predictions, SHAP analysis could only be applied on OmniConn RF trained on inhibitory scenario. Notably, the OmniConn model relied least on dip depth and most strongly on entropy, followed by KL divergence.(TIF)

S4 Fig CCG indicators distinguish successful and failed connectivity inference. Scatter plots of feature map means (FMMs) against four cross-correlogram indicators (peak height or dip depth, peak-to-noise or dip-to-noise ratio, normalized entropy, and KL divergence) across all baseline models for the connectivity inference task. Correctly predicted samples were clearly separated from failed predictions.
(TIF)

S5 Fig CCG indicators show substantial overlap between successful and failed weight inference predictions. Scatter plots of feature map means (FMMs) against four cross-correlogram indicators (peak height or dip depth, peak-to-noise or dip-to-noise ratio, normalized entropy, and KL divergence) across all baseline models for the synaptic weight inference task.
(TIF)

S6 Fig Stability of baseline model performance across fifty random initializations. (A) Coefficient of variation of overall model scores across 50 independent runs with different random seeds. (B) Standard deviation of test accuracies for each baseline model evaluated on all perturbation conditions over 50 runs in the connectivity-inference task. (C) Same as (B), but showing standard deviation of test losses in the synaptic-weight-inference task.
(TIF)

S7 Fig Representative CCGs and CCG indicator distributions under global firing-rate modulation regimes. (A) Distributions of CCG indicators across five firing-rate modulation regimes for excitatory (top row) and inhibitory (bottom row) connections. Columns show peak height/dip depth, peak-to-noise ratio, normalized entropy, and KL divergence within the central CCG window. The five modulation conditions were: Base (constant 20 Hz), Mild (15–20–25 Hz), Moderate (10–20–30 Hz), Strong (5–15–25–35 Hz), and Mean-shifted (5–15–30–45 Hz). (B) Representative excitatory (top row) and inhibitory (bottom row) CCGs from the five modulation regimes. Columns correspond to the Base, Mild, Moderate, Strong, and Mean-shifted conditions.
(TIF)

S8 FigPopulation-level topology schematics of the simulated network circuits.Population-level representations of the seven simulated network circuits using the graphical notation proposed by Senk, et al. [45]. Hexagons denote external stimulus populations ($X_{E}$,$X_{I}$), triangles denote recurrent excitatory populations ($S_{E}$), and circles denote recurrent inhibitory populations ($S_{I}$). Red arrows indicate excitatory projections, whereas blue circle-ended edges indicate inhibitory projections. Dashed edges represent probabilistic connectivity following a random fixed out-degree connectivity rule with autapses and multapses prohibited. Numbers inside nodes indicate population sizes, and edge labels indicate connection probabilities. Detailed neuron, synaptic, and simulation parameters for each circuit are provided in S3 Table. For visualization clarity, the full random fixed out-degree connectivity rule is explicitly illustrated only in the Network 1 schematic, whereas the remaining schematics display only connection fractions.(TIF)

S9 Fig Distribution of firing rates, ISIs CV and Fano factors. Histograms depict the distributions of mean firing rates (left), coefficients of variation of inter-spike intervals (ISIs CV, middle) and spike-count Fano factors (Fano Factor, right) across all excitatory (pink) and inhibitory (green) neurons in the simulated networks, and for the *in vitro* HD-MEA/patch-clamp dataset (top row). Detailed descriptions of the calculation of neuronal activity statistics for the *in vitro* HD-MEA/patch-clamp data are provided in the Methods section. Network 1 served as the unseen network for OmniConn models trained on pooled datasets across multiple networks. The four-network OmniConn was trained on networks 2–5, and the six-network OmniConn was trained on networks 2–7.
(TIF)

S10 Fig Raster plots of spiking activity from *in vitro* HD-MEA/patch-clamp datasets and synthetic circuits. Each panel shows spike times within a 0–4000 ms window. For synthetic circuits, 20 neurons (10 excitatory (red) and 10 inhibitory (blue)) were randomly selected; for the *in vitro* dataset, 20 neurons were randomly sampled from the set used for connectivity analysis. Synthetic networks span a wide range of dynamical regimes (from sparse to bursty to high-rate), whereas the *in vitro* data occupy a narrower, more heterogeneous but less strongly structured regime.
(TIF)

S11 Fig Cross-network generalization of CNN models trained on a single network and test on an unseen network. Each violin shows the distribution of CNN performance across 6 training networks, where each model was trained on CCGs from one network and tested on unseen CCGs from network 1. Different input representations are compared: raw CCGs (blue), scaled CCGs (red), and dual-channel CCGs (gray). (A) The left panel shows test accuracies for the connectivity-inference task, while the right panel shows test losses for the synaptic-weight-inference task. (B) Accuracy (left) and MCC (right) for connectivity inference on the HD-MEA with patch-clamp *in vitro* dataset. (C) Same model performance comparison across simulated network 1 and the HD-MEA experimental dataset. Left: Diverging bar plot showing the per-model accuracy difference (accuracy of network 1 minus accuracy of HD-MEA). Negative bars indicate models performing better on the simulated unseen dataset, and positive bars represent a relative advantage in the experimental dataset. Right: Dumbbell plot showing paired accuracies on both datasets for each model. Blue dots mark the accuracies of the model on simulated unseen dataset and red triangles represent model accuracies on the experimental dataset.
(TIF)

S12 Fig CCGs in *in vitro* HD-MEA/patch-clamp dataset. Among the 131 CCGs, 26 (black) represent experimentally defined connected pairs, whereas the remaining 105 (gray) correspond to unconnected pairs.
(TIF)

S13 Fig Comparison of binary and 3-class CNN performance on unseen simulated and experimental data. Each violin shows the distribution of CNN performance across 6 training networks, where each model was trained on CCGs from one network and tested on unseen CCGs from network 1. (A) Violin plots comparing accuracy distributions of binary (connection vs. no connection) and 3-class (excitatory, inhibitory, none) CNN classifiers on unseen simulated networks. (B) Violin plots showing model performance on the eANN dataset, which provides binary ground truth only. Binary CNNs show comparable or higher generalization accuracy in both settings, with lower variance across runs. These results support our decision to adopt a binary classification approach, which is more robust to class imbalance and aligns with the resolution of real experimental labels.
(TIF)

S1 TableBaseline parameter configurations and perturbation ranges for the 40  → 1 benchmarking experiments.This table summarizes the baseline model configurations and corresponding perturbation ranges used in the controlled 40 → 1 benchmarking framework. Each row represents a single baseline–perturbation combination. The first column indicates the baseline model name, the second column lists the parameters used during baseline training, and the remaining columns specify the parameters varied during perturbation testing. Fixed parameters are highlighted in red. Perturbed parameters are color-coded by category: mean level of background noise (μ; yellow), bursting probability (BP; blue), excitatory/inhibitory ratio (E/I; purple), and synaptic weight scaling (w; green).(XLSX)

S2 Table Benchmarking summary of ranking metrics for WeightCNN and ConnCNN across baseline models. (XLSX)

S3 Table Summary of network topology and simulation parameters. (XLSX)
